# Inflammation and Fibrosis in Progeria: Organ-Specific Responses in an HGPS Mouse Model

**DOI:** 10.3390/ijms25179323

**Published:** 2024-08-28

**Authors:** Peter Krüger, Moritz Schroll, Felix Fenzl, Eva-Maria Lederer, Ramona Hartinger, Rouven Arnold, Deniz Cagla Togan, Runjia Guo, Shiyu Liu, Andreas Petry, Agnes Görlach, Karima Djabali

**Affiliations:** 1 Epigenetics of Aging, Department of Dermatology and Allergy, TUM School of Medicine, Munich Institute of Biomedical Engineering (MIBE), Technical University of Munich (TUM), 85748 Garching, Germany; peter.krueger@tum.de (P.K.);; 2 Sanford Burnham Prebys Medical Discovery Institute, 10901 N Torrey Pines Rd, La Jolla, CA 92037, USA; 3 Experimental and Molecular Pediatric Cardiology, Department of Pediatric Cardiology and Congenital, Heart Diseases, German Heart Center Munich, Technical University Munich, 80636 Munich, Germany; 4 DZHK (German Centre for Cardiovascular Research), Partner Site Munich Heart Alliance, 80636 Munich, Germany

**Keywords:** Hutchinson–Gilford Progeria Syndrome, lamin A, progerin, fibrosis, inflammation, aging, senescence

## Abstract

Hutchinson–Gilford Progeria Syndrome (HGPS) is an extremely rare genetic disorder that causes accelerated aging, due to a pathogenic variant in the LMNA gene. This pathogenic results in the production of progerin, a defective protein that disrupts the nuclear lamina’s structure. In our study, we conducted a histopathological analysis of various organs in the Lmna^G609G/G609G^ mouse model, which is commonly used to study HGPS. The objective of this study was to show that progerin accumulation drives systemic but organ-specific tissue damage and accelerated aging phenotypes. Our findings show significant fibrosis, inflammation, and dysfunction in multiple organ systems, including the skin, cardiovascular system, muscles, lungs, liver, kidneys, spleen, thymus, and heart. Specifically, we observed severe vascular fibrosis, reduced muscle regeneration, lung tissue remodeling, depletion of fat in the liver, and disruptions in immune structures. These results underscore the systemic nature of the disease and suggest that chronic inflammation and fibrosis play crucial roles in the accelerated aging seen in HGPS. Additionally, our study highlights that each organ responds differently to the toxic effects of progerin, indicating that there are distinct mechanisms of tissue-specific damage.

## 1. Introduction

HGPS was first discovered as a distinct medical condition in 1886 by Jonathan Hutchinson and Hastings Gilford; the latter coined the term “progeria” for this disease [1,2]. Both surgeons reported independently about this disease and described the observed premature aging characteristics like hair loss, growth retardation, and dermal abnormalities [1,2]. The progeroid phenotype was characterized extensively in the literature adding micrognathia, increased osteolysis, prominent scalp veins, joint abnormalities, lipodystrophy and atherosclerosis, a high-pitched voice, and early death from coronary artery disease or stroke to the list of physiological malfunctions [3,4,5]. Novel therapeutics are emerging for the treatment of orphan diseases associated with the premature aging phenotype. One of those orphan drugs, called Lonafarnib, was the first of its kind to be FDA-approved for the treatment of Hutchinson–Gilford Progeria Syndrome (HGPS) and other laminopathies (HGPS: OMIM: #176670) [6] highlighting the progress in the field of premature aging diseases.

HGPS is caused by a silent single nucleotide substitution mutation at C.1824C > T in exon 11 of the LMNA gene, introducing a lamin-A specific cryptic splice site not affecting the lamin-C transcript [7,8,9]. The precursor prelamin A protein normally undergoes a series of rapid post-translational modifications involving the addition of a farnesyl group to the cysteine residue at the C-terminus CAAX motif, and then cleavage of the last three amino acids (AAX) by the endoprotease ZMPSTE24 or RCE1 exposing the last cysteine for carboxymethylation [10]. During the last step of prelamin A maturation, ZMPSTE24 cleaves off the last 15 amino acids and as such, removes the farnesyl group [11]. However, in the mutant prelamin A, progerin, expressed in HGPS cells, this last maturation step does not occur as the ZMPSTE24 cleavage site is lost and therefore progerin remains permanently farnesylated and carboxymethylated [12,13]. Progerin’s inability to undergo the complete posttranslational modification causes its accumulation at the inner nuclear envelope within the nuclear lamina over time [14,15]. This progerin build-up induces structural disruptions causing cellular defects including DNA damage, epigenetic alterations chromatin reorganization, and gene expression reprogramming [14,16].

Progerin’s cytotoxic effect and the resulting premature aging phenotype calls for in-depth in vivo examination of various organs to further understand the HGPS pathologies and establish histological readouts to track the efficacy of novel therapeutic approaches. Progerin-induced defects have been intensively investigated in various cell models and several mouse models of HGPS have also been created and used in different laboratories [17,18,19,20]. One of the most characterized progerin mouse models mimicking the human HGPS condition is the Lmna^G609G/G609G^ mice from Carlos López-Otín’s lab [21]. This model is a knock-in mouse strain carrying the HGPS pathogenic variant, where the wildtype mouse Lmna gene is replaced with a mutant allele that carries the c.1827C > T; p.Gly609Gly variant, which is equivalent to the HGPS c.1824C > T; p.Gly608Gly mutation in the human LMNA gene [21,22]. Homozygous progerin (Lmna^G609G/G609G^) mice are showing growth retardation as early as 3 weeks, reduced weight, kyphosis, and vascular disease as well as metabolic symptoms including hypoglycemia and reduced insulin-like growth factor levels [22,23].

The Lmna^G609G^ mouse model was extensively characterized in a long-term breeding study and showed nuclear abnormalities, reduced subcutaneous fat, increased β-galactosidase activity in liver and kidney, as well as reduced thymus and spleen size [21,22]. Moreover, they exhibit reduced bone mineral density, blood plasma hypoglycemia, reduced leptin and increased adiponectin concentrations, and loss of vascular smooth muscle cells (VSMCs) in the aortic media [24,25]. The cardiovascular alterations include aortic stiffness, media thickness reduction, collagen deposition in the media, and adventitia and elastin fiber waving changes [26,27]. Furthermore, Benedicto et al. demonstrated that apart from VSMC loss, endothelial cells (ECs) also play a crucial role in disease progression. Defective endothelial mechanotransduction contributes to the pathological cardiovascular phenotype, including inflammation, vessel stiffening, and vessel dysfunction [28]. Lifespan studies from different laboratories indicate that the homozygous Lmna^G609G/G609G^ mouse dies approximately at the age of 3.5 months [22].

Heterozygous and homozygous animals have been further characterized. Female mutant mice are born less frequently (female 1:1.13 male) and around 50% of the weaned pups are heterozygous and 20% homozygous coming from het-het parent animals indicating a non-mendelian birth rate across genotypes [21]. Atrophy of subcutaneous fat in the skin, fibrosis and reduced number of hair follicles, and VSMC loss in the ascending aorta were reported [21]. Moreover, skeletal muscle atrophy and muscle leucocyte infiltration were also reported [21]. Apart from this, mitochondrial alterations have been described in dermal fibroblasts, cardiomyocytes, and skeletal muscle [21,29,30].

A particularly fascinating aspect of HGPS pathology is its rapid aging progression and the associated decline in tissue functionality. The role of progerin expression as a key factor driving these changes still remains to be further investigated. Importantly, inflammation and fibrosis have been observed in arterial lesions of HGPS patients [31]. Moreover, nuclear factor kappa-light-chain-enhancer of activated B cells (NF-κB) signaling, a key player in immune response, inflammation, and cell survival is activated, and high levels of pro-inflammatory cytokines are detected in mouse models of HGPS [32]. This suggests a critical connection between progerin expression and inflammation in triggering accelerated aging. Additionally, another study showed a significant upregulation of several pro-inflammatory cytokines including Interleukin-6 (IL-6) and tumor necrosis factor alpha (TNFα) that are known to induce a chronic inflammatory state via feed-forward regulatory signaling, which impacts remote cells and tissues [33].

Furthermore, the role of IL-6 revealed that its inhibition, via the use of tocilizumab (a neutralizing antibody targeting IL-6 receptors), countered progeroid characteristics in both HGPS fibroblasts and progeroid mice [34]. The administration of tocilizumab effectively reduced the build-up of progerin, rectified nuclear envelope and chromatin abnormalities, and mitigated the hyperactivated DNA damage response. Importantly, in vivo administration of tocilizumab was shown to decrease aortic lesions, curb adipose tissue dystrophy, and delay the onset of lipodystrophy and kyphosis, in Lmna^G609G/G609G^.

To further interrogate the potential involvement of inflammation and tissue fibrosis in the development of HGPS pathologies, we examined the pathology of Lmna^G609G/G609G^ homozygous mice in this study, by analyzing the presence of inflammation and fibrosis in various organs. By examining the skin, aorta, liver, kidney, thymus, spleen, heart, lung, and muscle at the end of Lmna^G609G/G609G^ lifespan and comparing them with their wildtype Lmna^+/+^ littermates, a comprehensive analysis of the impact of aging on these organs was determined. The findings reveal that aging affects each organ distinctly, with vascular fibrosis being a common issue in HGPS mice across all examined organs. However, the extent and nature of fibrosis varied significantly between different organs. Notably, the skin exhibited the most severe fibrotic damage, while the spleen’s primary defect was the dysregulation of the marginal zone (MZ). This study highlights the diverse tissue-specific susceptibility to fibrotic and inflammatory events in different organs within this Lmna^G609G/G609G^ mouse model.

## 2. Results

### 2.1. Skin

**Figure 1:**
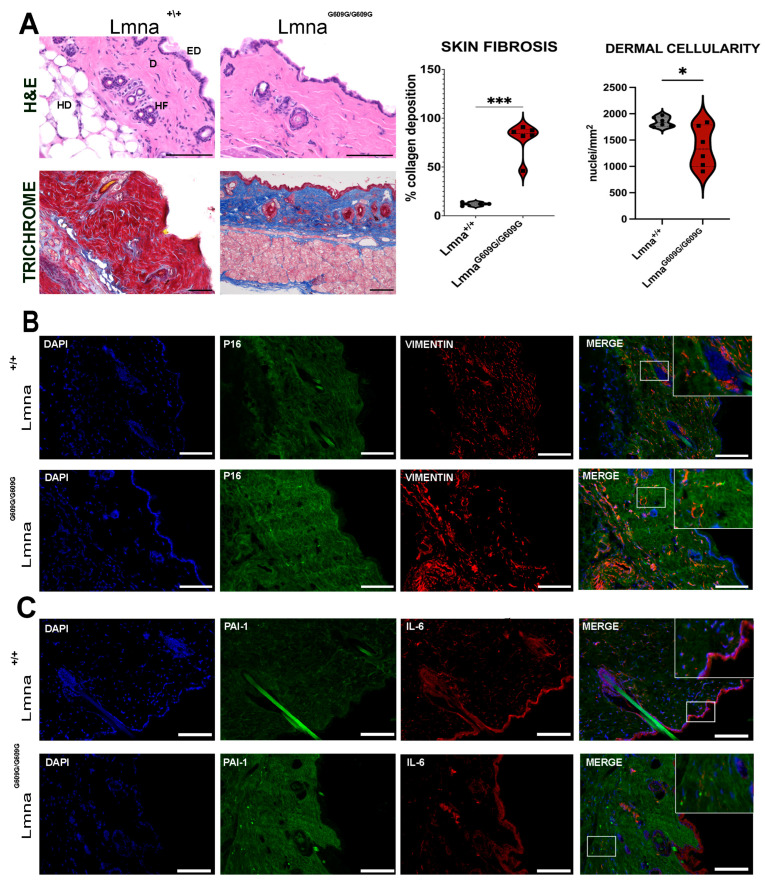
Histological and Immunofluorescent characterization of dorsal skin pathology in Lmna^G609G/G609G^ mice compared to wildtype C57B/6 mice (EP = epidermis; D = dermis; HD = hypodermis; HF = hair follicle) using a Keyence BZ-X810 microscope and an Axio Imager D2 (Carl Zeiss, Oberkochen, Germany): (**A**) Hematoxylin and Eosin (HE) staining of dorsal skin depicting the epidermis, dermis, and subdermis skin layers. Epidermal layer thickness remained constant between progerin and wildtype mice, forming a single-cell border. Dermis show disorganized collagen fibers. Hypodermal region and hair follicles showed no difference between genotypes. Dermal cellularity was reduced in Lmna^G609G/G609G^ mice (scale bar = 100 µm; n = 6; * *p* < 0.05). Masson’s Trichrome staining of wildtype and Lmna^G609G/G609G^ dorsal skin cryotome sections (6 µm). Collagen is stained in blue, cytoplasm and keratin are stained in red. In Lmna^G609G/G609G^ mouse skin, collagen deposition was highly increased compared to the Lmna^+/+^ cohort. (scale bar = 100 µm; n = 6; *** *p* < 0.0005) (**B**) Immunofluorescent staining of dorsal skin sections with anti-vimentin [35] and anti-p16 (green) antibodies. Vimentin signal was increased in Lmna^G609G/G609G^ in the hypodermal layer and dermal signal was reduced. Anti-p16 signal was stronger in Lmna^G609G/G609G^ mice. We observed that p16 was accumulating in the dermis. This accumulation highlights discrete focal points of increased antibody binding indicative of sites associated with increased cellular senescence (n = 3; scale bar = 100 µm). Zoom-box was added and is depicted in white brackets. (**C**) Immunofluorescent staining of dorsal skin with anti-PAI-1 (green) and anti-IL-6 [35] antibodies. The PAI-1 signal was increased in Lmna^G609G/G609G^ skin with focal points increased with antibody binding. IL-6 signal did not show drastic differences between wildtype and mutant samples (n = 3; scale bar = 100 µm). Zoom-box was added and is depicted in white brackets.

We conducted a comprehensive evaluation of mouse dorsal skin samples, employing Hematoxylin and Eosin staining for visualization (Figure 1A). Our analysis revealed a notable reduction in cellularity within the dermal layer of Lmna^G609G/G609G^ mice compared to their Lmna^+/+^ (WT) counterparts. Additionally, male mutant mice exhibited a noticeable decrease in dermal thickness (Figure S1). Most strikingly, our investigation unveiled a substantial increase in fibrotic tissue deposition within the progeria mouse dermis (Figure 1A). Assessment of p16 signal intensity in the skin demonstrated a marked increase in Lmna^G609G/G609G^ mice (Figure 1B). Conversely, there were observable alterations in the distribution of vimentin, with a pronounced decrease in vimentin expression within the dermal layer of mutant mice (Figure 1B). The dermis in mutant dorsal skin showed areas lacking a vimentin signal, whereas the WT samples showed an even distribution of vimentin in the dermal area. In contrast, the hypodermal region exhibited a stronger Vimentin signal in Lmna^G609G/G609G^ mice (Figure 1B). Plasminogen activator inhibitor 1 (PAI-1/serpine-1) signal was observed to be increased in mutant mice, showing beacons of accumulated signal in dermal regions. IL-6 signal showed no changes between the two genotypes (Figure 1C).

Altogether, these findings indicate alterations in Lmna^G609G/G609G^ dermis with increased collagen deposits, loss of cellularity, and accumulation of PAI-1 indicating skin fibrosis.

### 2.2. Aorta

**Figure 2:**
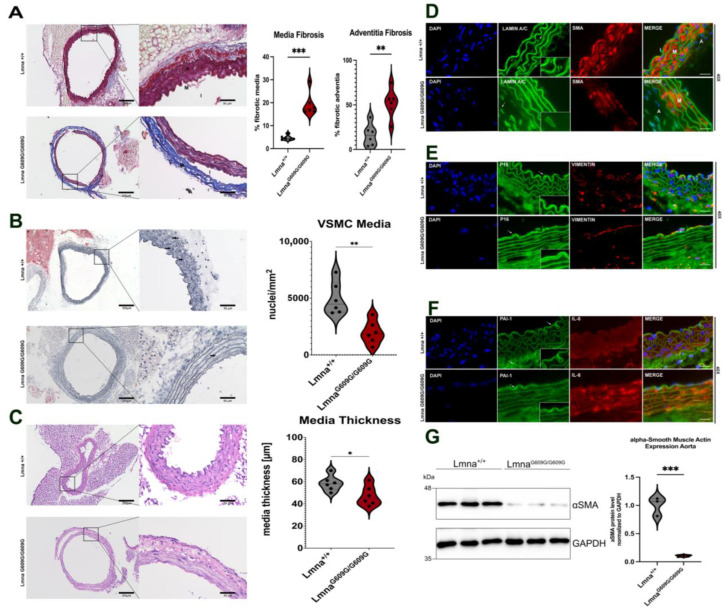
Histological and Immunofluorescent characterization of thoracic aorta pathology in Lmna^G609G/G609G^ mice compared to wildtype C57B/6 mice. (M = media; A = adventitia, I = intima). Histopathological and immunofluorescent staining at 10× and 40× magnification using the Keyence BZ-X810 microscope and an Axio Imager D2: (**A**) Media and adventitia fibrosis was assessed using Masson’s Trichrome staining. Significant increase was detected in the adventitia and inside the media in between the elastin fibers (n = 6; *** *p* < 0.0005; scale bar = 200 µm (10×)/50 µm (40×)). (**B**) Oil Red O staining counterstained with Hematoxylin to visualize nuclei in aortic media. Media cellularity was found significantly reduced in Lmna^G609G/G609G^, no lipid staining was observed except in periaortic adipose tissue surrounding the aorta (n = 4; ** *p* < 0.005; scale bar = 200 µm (10×)/50 µm (40×); arrow = nuclei). (**C**) Media thickness of thoracic aorta was compared between Lmna^+/+^ and Lmna^G609G/G609G^ mice. Lmna^G609G/G609G^ mice had a reduced media thickness compared to wildtype animals (n = 6; * *p* < 0.05; scale bar = 200 µm (10×)/50 µm (40×)). (**D**) LaminA/C (green), αSMA [35], and 4′,6-diamidino-2-phenylindole (DAPI) staining in aorta; αSMA staining together with DAPI indicates loss of VSMC in Lmna^G609G/G609G^ media. (n = 3; scale bar = 20 µm). Zoom-box was added to show Lamin nuclear rim staining. (**E**) p16 (green), Vimentin [35], and DAPI (blue) staining; p16 indicating senescence was predominantly found in the intima and media of Lmna^G609G/G609G^ mice. Vimentin signal was lost in media of Lmna^G609G/G609G^ mice but was detected equally in intima and adventitia of both wildtype and mutant mice (scale bar = 20 µm). Zoom-box was added to show specificity of p16 staining at the intima. White arrows indicate location of p16 staining. (**F**) Serpine-1 (PAI-1) (green), IL-6 [35], and DAPI (blue) staining. PAI-1 signal visible mostly in adventitial region and epidermal layer of the intima. PAI-1 signal was increased in endothelial cells of the aortic intima of Lmna^G609G/G609G^ mice (scale bar = 20 µm). White arrows indicate PAI-1 staining location. Zoom-box was added to show staining specificity at the intima. (**G**) Western blot detection of αSMA in Lmna^G609G/G609G^ and Lmna^+/+^ mice showing significantly reduced levels of this protein in mutant aortas ** *p* < 0.0005).

The thoracic aorta was assessed using trichrome staining to examine changes in fibrotic tissue. A significant increase in collagen content was observed in the Lmna^G609G/G609G^ media and adventitia of the aorta (Figure 2A,B). Additionally, there was a substantial reduction in vascular smooth muscle cells in the media, and the elastin fibers were more linear in Lmna^G609G/G609G^ mice compared to those of wildtype, indicating elastin disruption (Figure 2B). Furthermore, the media thickness in Lmna^G609G/G609G^ mice was notably reduced (Figure 2C). Immunofluorescent staining and Western blot analyses for α-smooth muscle actin (α SMA) confirmed the previously reported loss of vascular smooth muscle cells [22,36] (Figure 2D,G). In Lmna^G609G/G609G^ mice, there was a complete depletion of the vimentin signal in the media, with no changes observed in the intima and adventitia (Figure 2D,E). Elevated p16 levels were observed in the endothelial layer of the intima (Figure 2E). Hence, PAI-1, known to accumulate in fibrotic tissues [37], was increased in the endothelial layers of the intima and adventitia of Lmna^G609G/G609G^ mice compared to Lmna^+/+^ mice (Figure 2F). IL-6 levels were also markedly increased in the media, indicating the presence of inflammation in the Lmna^G609G/G609G^ mouse aorta (Figure 2F).

Collectively, these results highlight severe alterations in the ascending aorta of Lmna^G609G/G609G^ mice, indicative of inflammation and fibrosis.

### 2.3. Muscle

**Figure 3:**
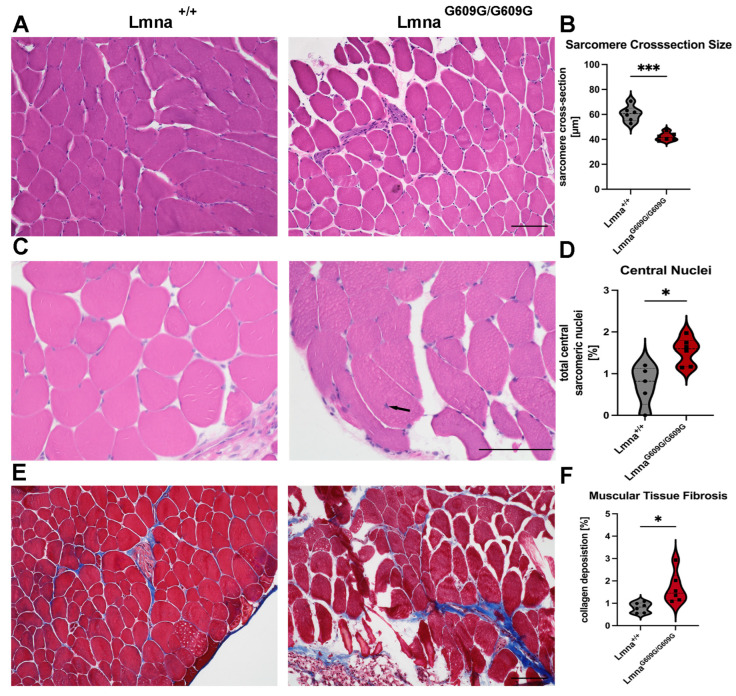
Histological and Immunofluorescent characterization of muscular (gastrocnemius) pathology in Lmna^G609G/G609G^ mice compared to wildtype C57B/6 mice: (**A**) H&E staining used to quantify sarcomere diameter in transversal gastrocnemius muscle cuts showing reduced size in Lmna^G609G/G609G^ mice (n = 6; *** *p* < 0.0005; scale bar = 100 µm). (**B**) Graphic showing sarcomere size cross-section variation comparing Lmna^+/+^ and Lmna^G609G/G609G^ mice. (**C**) Nuclear position was assessed in HE-stained pictures showing elevated levels of central nuclei mislocalization in Lmna^G609G/G609G^ mice. Dark arrow shows location of a central nucleus in Lmna^G609G/G609G^ mice (n = 6; * *p* < 0.05, scale bar = 100 µm). (**D**) Graphic showing central nucleus occurrence comparing Lmna^+/+^ and Lmna^G609G/G609G^ mice. (**E**) Masson’s Trichrome staining indicating muscular fibrosis. Fibrosis was increased in muscular vasculature and in the perimuscular space of Lmna^G609G/G609G^ mice (n = 6; * *p* < 0.05; scale bar = 100 µm). (**F**) Graphic showing percentage of collagen deposition in muscular tissue comparing Lmna^+/+^ and Lmna^G609G/G609G^ mice.

The evaluation of the size of 1200 sarcomeres (600 wildtypes and 600 homozygous) indicated a significant reduction in sarcomere size in Lmna^G609G/G609G^ mice, with sarcomere cross-sectional dimensions measuring an average of 60 µm in wildtype and 40 µm in mutant mice (Figure 3A,B). To assess muscle pathology, we conducted an analysis of sarcomere nucleus localization, revealing a significant increase in central sarcomere nuclei in homozygous animals (1.5%) compared to wildtype animals (0.9%) (Figure 3C,D).

Furthermore, increased fibrosis in muscle tissue of Lmna^G609G/G609G^ mice was observed, compared to wildtype animals (Figure 3E,F). The total collagen signal increased from 0.7% in wildtype to 1.6% in Lmna^G609G/G609G^ mice. The collagen signal was primarily localized in the perimysium and not within the sarcomeres of Lmna^G609G/G609G^ mice (Figure 3E,F). However, vimentin and αSMA staining showed no significant differences between wildtype and homozygous samples (Figure S2). However, the CD68 signal that detects the presence of macrophages showed no changes between muscle sections from wildtype and Lmna^G609G/G609G^ mice (Figure S3). Additionally, we noted increased expression of Pax7 in mutant mice, with the signal predominantly situated in the sarcomere periphery (Figure S3). This finding suggests a response to muscle damage or a compensatory mechanism for impaired muscle function in the mutant mice. This observation will require further investigation in the future.

### 2.4. Lung

**Figure 4:**
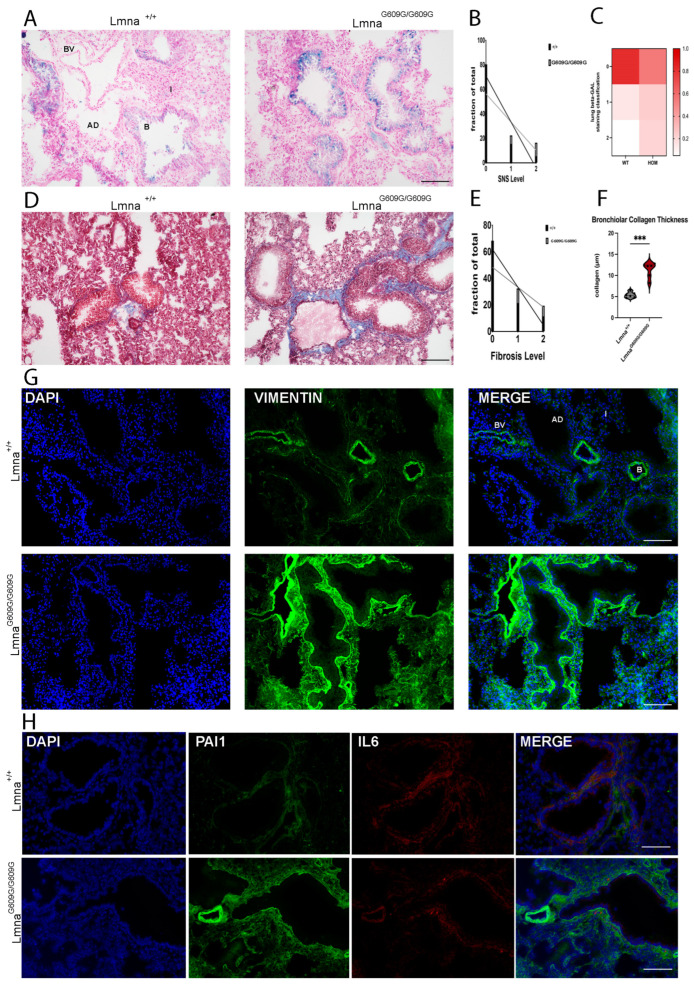
Histological and Immunofluorescent characterization of lung pathology in Lmna^G609G/G609G^ mice compared to wildtype C57B/6 mice. Histopathological and immunofluorescent staining at 10× and 40× magnification using the Keyence BZ-X810 microscope and an Axio Imager D2 (B = bronchiole, AD = alveolar duct; I = Interstitium; BV = blood vessel): (**A**) Increased β-Galactosidase activity in Lmna^G609G/G609G^ reveals elevated number of senescent cells in lung. Senescence detected mostly in club cells of bronchiole (n = 6; scale bar = 100 µm). (**B**) Graphic showing fraction of total lung bronchiolar senescence for each level of senescence comparing Lmna^+/+^ and Lmna^G609G/G609G^ mice. (**C**) Heatmap depicting the fraction of total each genotype displays each level of senescence. (**D**) Masson’s Trichrome staining indicating pulmonary fibrosis. Lmna^G609G/G609G^ mice display elevated levels of fibrosis around bronchioles and pulmonary vasculature (n = 6; scale bar = 100 µm). (**E**) Graphic showing fraction of total lung collagen deposition for each level of fibrosis comparing Lmna^+/+^ and Lmna^G609G/G609G^ mice. (**F**) Graphic showing collagen rim thickness (µm) around bronchiole comparing Lmna^+/+^ and Lmna^G609G/G609G^ mice (n = 6; *** *p* < 0.0005). (**G**) IF staining showing increased Vimentin signal in mutant mice (n = 3, scale bar = 200 µm). (**H**) IF staining showing increased Serpine-1 (PAI-1) signal in bronchioles and in close proximity to vasculature of Lmna^G609G/G609G^ mice but no change in IL-6 (n = 3, scale bar = 100 µm).

We assessed HGPS mouse lung tissues focused on the physiology of alveoli and bronchioles, with an emphasis on interstitial fibrotic changes. The predominant changes were observed in the bronchioles of Lmna^G609G/G609G^ mice (Figure 4A). Senescence-associated beta-galactosidase staining showed an increased number of senescent cells in lung sections of Lmna^G609G/G609G^ mice (Figure 4A–C). Tissue pathology was graded on a scale from 0 to 2, with 0 representing no senescence/fibrosis, 1 indicating pre-senescent or pre-fibrotic bronchioles, and 2 representing senescent bronchioles or strong bronchiolar fibrosis (Figure 4D–F).

An increased level of pre-senescent and senescent bronchioles was detected in Lmna^G609G/G609G^ mice (Figure 4A–C). Collagen deposition was frequently observed around Lmna^G609G/G609G^ bronchioles (Figure 4D–F). The interstitial fibrosis in the lung tissue was not observed and therefore only bronchiolar fibrosis was assessed (Figure 4D–F). Hence, the vimentin signal was strongly elevated in Lmna^G609G/G609G^ mice lung tissue (Figure 4G). These results suggest a hyperactivation and proliferation of fibroblasts and enhanced collagen synthesis in Lmna^G609G/G609G^ lung tissue, a process contributing to fibrosis (Figure 4G). Furthermore, we observed an increase in PAI-1 at bronchioles and vascular tissue of Lmna^G609G/G609G^ mice (Figure 4H). IL-6 signal showed no obvious changes between wildtype and mutant mouse tissues (Figure 4H).

### 2.5. Liver

**Figure 5:**
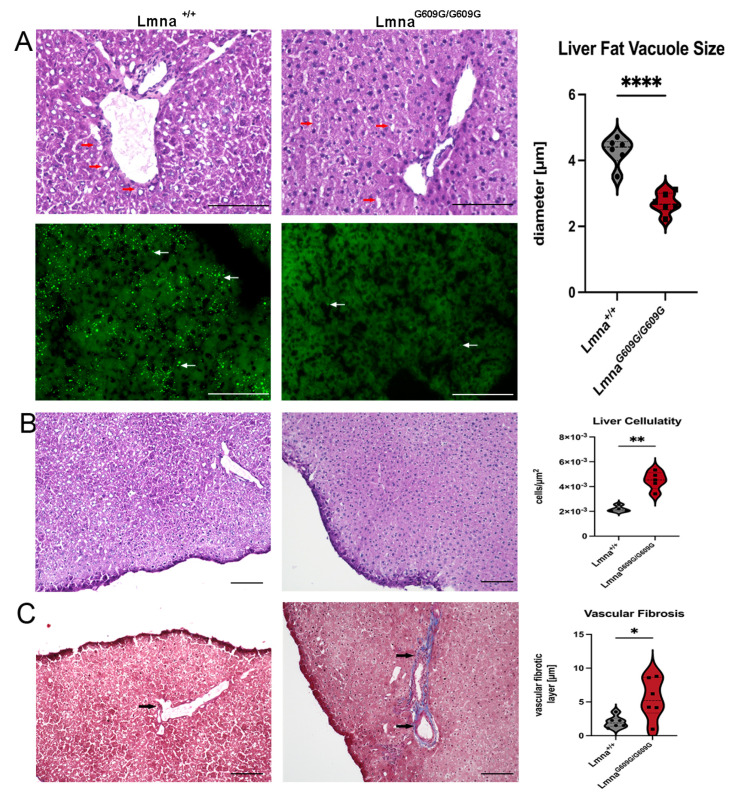
Histological and Immunofluorescent characterization of liver pathology in Lmna^G609G/G609G^ mice compared to wildtype C57B/6 mice. Histopathological and immunofluorescent staining at 10× and 40× magnification using the Keyence BZ-X810 microscope (scale bar = 100 µm): (**A**) HE staining showing variety in liver fat vesicle size between Lmna^+/+^ and Lmna^G609G/G609G^ mice. Significant reduction in vacuole size in Lmna^G609G/G609G^ mice (n = 6; **** *p* < 0.00005). Fat vesicles were also visualized by Bodipy staining (n = 3; arrows = lipid vesicles). (**B**) Quantification of liver cellularity. Lmna^G609G/G609G^ mice display increased cellularity (n = 4 (WT); n = 5 [25]); ** *p* < 0.005). (**C**) Masson’ Trichrome staining indicating elevated vascular fibrosis in hepatic tissue of Lmna^G609G/G609G^ mice. We did not observe significant interstitial fibrosis in wildtype or diseased animal livers (n = 6; * *p* < 0.05).

In the livers of Lmna^G609G/G609G^ mice, a significant reduction in the size of fat vacuoles was observed, as indicated by both HE and Bodipy staining (Figure 5A). We quantified vacuole size by measuring 6891 vacuoles from 12 experimental animals (6 wildtype and 6 homozygous) (Figure 5A, left panel). Hepatic cellularity was significantly increased in Lmna^G609G/G609G^ animals (Figure 5B). This increased cellularity indicates atrophy of the hepatocytes which appears as a reduction in cell size and reduction in total liver size (Figure S4) The phenomenon of hepatic atrophy is often seen in physiological aging in humans and is associated with a reduced functionality of the liver [38].

For the assessment of fibrosis in the liver, we used the Ishak score [39]. Wildtype mice primarily scored 0–1, whereas homozygous mice mostly scored 2 (Figure S5). Although the Ishak score is a good indication of fibrotic changes in the liver, nevertheless we pursued a more quantitative approach by measuring the diameter of collagen-stained areas around vascular tissue in the liver and statistically evaluated those values (Figure 5C). Portal fibrosis was observed in a minority of wildtype samples, with prominent portal fibrosis evident in homozygous animals. Fibrosis of the hepatic septum was not observed, and there were no discernible differences in vimentin staining and αSMA between wildtype and mutant samples (Figure S6). These findings indicate that the livers of the mutant mice at the end of their lifespan showed significant hepatic atrophy, increased cellularity, and enhanced fibrosis, indicative of reduced liver functionality.

### 2.6. Kidney

**Figure 6:**
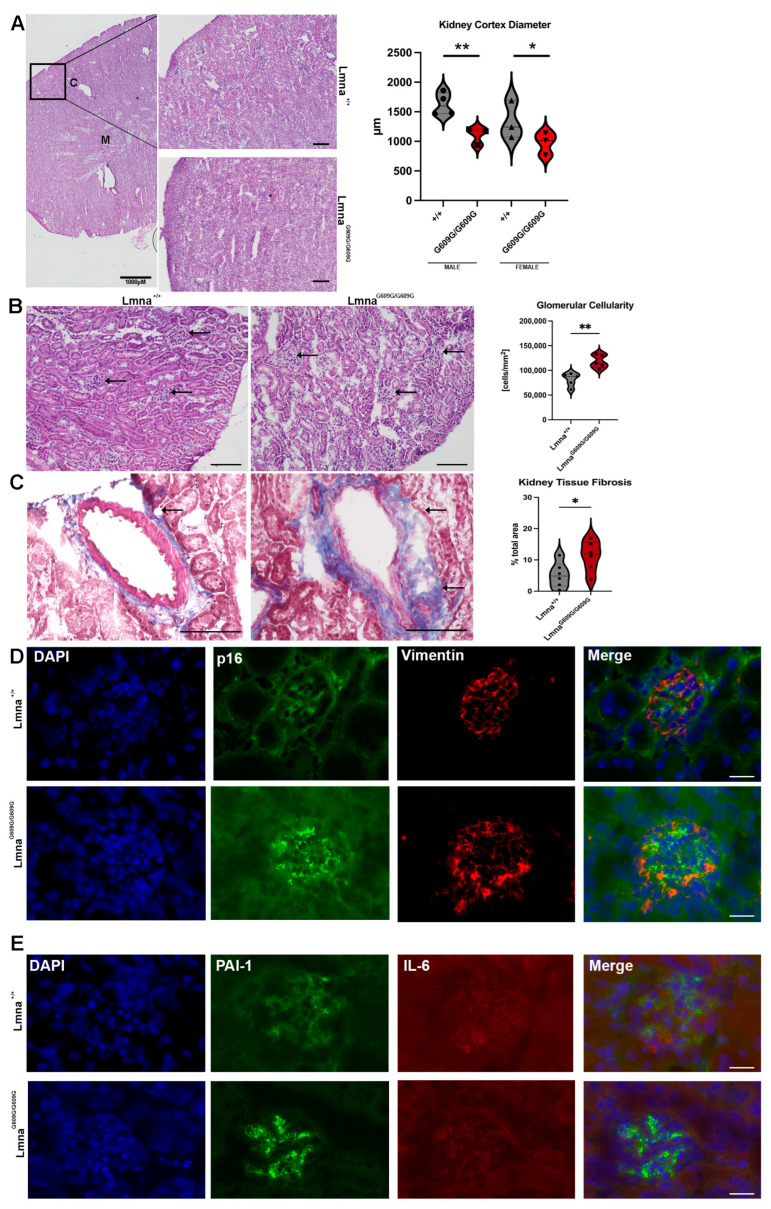
Histological and Immunofluorescent characterization of kidney pathology in Lmna^G609G/G609G^ mice compared to wildtype C57B/6 mice (C = cortex; M = medulla) using a Keyence BZ-X810 microscope and an Axio Imager D2: (**A**) HE staining used to quantify renal cortex thickness. The renal cortex displays significantly reduced radius in Lmna ^G609G/G609G^ mice (n = 6; * *p* < 0.05; ** *p* < 0.005); scale bar = 100 µm, arrow indicating collagen deposition). (**B**) HE staining used to assess renal glomeruli leukocyte infiltration by observation of increased total. Significant increase in cell number indicates leukocyte infiltration. Glomeruli are marked with black arrows. (n = 5; ** *p* < 0.005); scale bar = 100 µm). (**C**) Masson’s Trichrome staining used to visualize vascular and interstitial fibrosis. Interstitial fibrosis was not observed but vascular fibrosis was significantly increased in diseased mice (n = 6; * *p* < 0.05; scale bar = 100 µm; arrow indicating glomeruli). (**D**) p16 (green), Vimentin [35], and DAPI staining showing glomeruli of Lmna^G609G/G609G^ and Lmna^+/+^ mice. Vimentin signal was unchanged but p16 signal was elevated in glomeruli of Lmna^G609G/G609G^ mice (scale bar = 20 µm). (**E**) Serpine-1 (PAI-1) (green), IL-6 [35], and DAPI staining of mutant and C57B/6 glomeruli. IL-6 signal is unchanged but PAI-1 signal is increased in Lmna^G609G/G609G^ mice (scale bar = 20 µm).

At the endpoint of their lifespans, Lmna^G609G/G609G^ mice displayed smaller kidneys compared to age-matched wildtype animals (Figure S4). The cortex diameter was significantly larger in wildtype animals than in mutants (Figure 6A). Additionally, there was a significantly increased number of cells in the glomeruli of Lmna^G609G/G609G^ mice (Figure 6B). While interstitial fibrosis and collagen deposition around renal ducts and glomeruli were not observed, there was a significant increase in collagen deposition proximal to the blood vessels in Lmna^G609G/G609G^ mice (Figure 6C). Minimal evidence of debris, kidney stones, and calcification was noted, with only one crystal structure observed in a male homozygous animal (Figure S7) out of 12 examined. Glomerulonephritis was indicated by the infiltration of glomeruli by cells, although glomerular rim enlargement was not pronounced (Figure 6B). Vimentin signal in Lmna^G609G/G609G^ was more pronounced at the periphery of the glomerulus and p16 was strongly increased in mutant mice (Figure 6D and Figure S8). IL-6 signal was unchanged comparing the two genotypes, but PAI-1 signal was increased in Lmna^G609G/G609G^ (Figure 6E). Collectively, these observations indicate that mutant mice at their end point exhibited significant renal abnormalities, including smaller kidneys, increased glomerular cell numbers, enhanced collagen deposition around blood vessels, and elevated markers of cellular stress and senescence compared to wildtype animals of similar age.

### 2.7. Spleen

**Figure 7:**
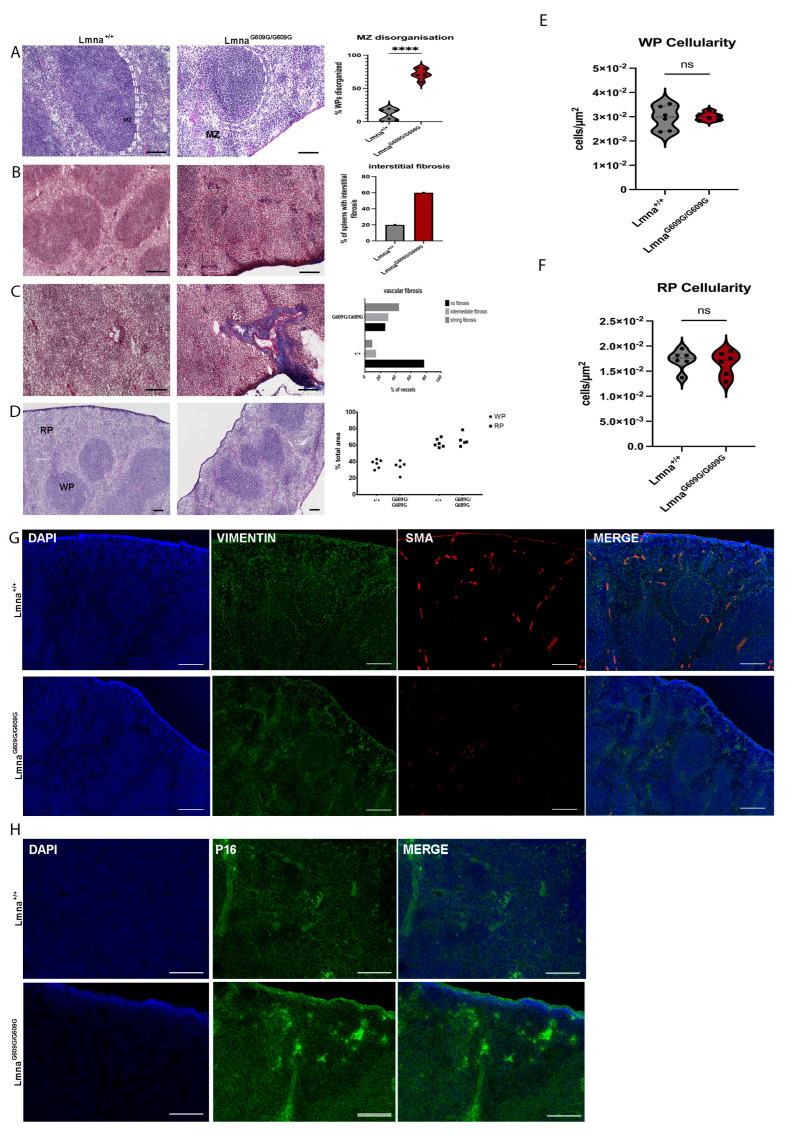
Histological and Immunofluorescent characterization of splenic pathology in Lmna^G609G/G609G^ mice compared to wildtype C57B/6 mice using a Keyence BZ-X810 microscope and an Axio Imager D2. (WP = white pulp, RP = red pulp, MZ and dashed white lines indicating marginal zone, C = splenic capsule). Histopathological and immunofluorescent staining at 10× and 40× magnification using the Keyence BZ-X810 microscope: (**A**) H&E staining of spleen showing WP, RP, and spleen capsule. Mutant animals exhibit MZ disorganization at the border between RP and WP (n = 6; **** *p* < 0.00005; scale bar = 100 µm). (**B**) Masson’s Trichrome staining indicating elevated interstitial and vascular fibrosis in the spleen of Lmna^G609G/G609G^ mice (n = 6; scale bar = 100 µm). (**C**) Masson’s Trichrome staining indicating increased vascular fibrosis in the spleen (n = 6; scale bar = 100 µm). (**D**) Ratio of total WP and RP area was compared between mutant and wildtype cohorts showing no difference. (**E**) Comparing WP cellularity between Lmna^G609G/G609G^ and wildtype mice. (**F**) Comparing WP cellularity between Lmna^G609G/G609G^ and wildtype mice. (**G**) Vimentin (green), αSMA [35], and DAPI staining ECM producing myofibroblasts, vascular smooth muscle cells, and nuclei in the spleen. Vimentin staining reveals a distinct border co-localized with the MZ separating the WP and RP showcasing the structural integrity in Lmna^+/+^ spleens, while its disorganization is evident in the Lmna^G609G/G609G^ mouse spleen. αSMA was reduced in Lmna^G609G/G609G^ mice. (**H**) DAPI and p16 staining of spleen tissue. p16 signal is increased in samples of Lmna^G609G/G609G^ mice displaying increased intensity mostly inside the WP region indicating increased senescence.

Previous research has demonstrated splenic retardation in this mouse model of progeria [22]. Spleens of Lmna^G609G/G609G^ showed a decline in spleen size with growing age (Figure S4). Disorganization of the marginal zone (MZ) within the spleen was also observed, affecting around 70% of all RP-WP regions (6 wildtype and 6 homozygous animals) of Lmna^G609G/G609G^ animals but only about 10% in Lmna^+/+^ mice (Figure 7A). However, collagen deposits were notably increased in both the red pulp and white pulp of Lmna^G609G/G609G^ spleens, with fibrotic lesions observed in approximately 60% of Lmna^G609G/G609G^ mice and in 20% of wildtype spleens (Figure 7B). Additionally, fibrotic changes were pronounced in the splenic vasculature, with approximately 75% of blood vessels in homozygous animals exhibiting collagen deposition compared to 20% in wildtype mice (Figure 7C).

Despite the reduced size of the spleen, we did not observe significant changes in the red pulp (RP) or white pulp (WP) cellularity (Figure 7E,F). Moreover, the RP-WP ratio remained unaltered, indicating equal tissue degradation across various splenic tissue regions (Figure 7D,F,G). The mutant spleen displayed an increased progerin/Lamin C ratio compared to the wildtype, indicating an accumulation of progerin in the nuclei of this tissue. Except for the heart and the spleen, the other tissues did not show a change in this progerin/Lamin C ratio (Figure S9).

Vimentin staining in the red pulp was increased in Lmna^G609G/G609G^ animals, aligning with the previously reported collagen deposition primarily in this area (Figure 7G). Furthermore, elevated vimentin accumulation was observed in and around blood vessels (Figure 7G). The structural integrity of the RP and WP, regulated by mesenchymal cells, is vital for the spleen’s functionality as a mesenchymal-derived organ. Immunofluorescence of vimentin revealed that wildtype mice exhibited stronger mesenchymal cell support for the MZ, forming a clear border between RP and WP, while this vimentin signal was notably weaker in Lmna^G609G/G609G^ mice. αSMA staining illustrated the loss of vascular smooth muscle cells (VSMCs) in blood vessels and the capsule of mutant mouse spleens (Figure 7G). Additionally, we observed a marked increase in p16 staining in the G609G mutant spleen samples. The staining is mostly localized inside the white pulp of the spleen indicating an enhanced cellular senescence in this region (Figure 7H).

### 2.8. Thymus

**Figure 8:**
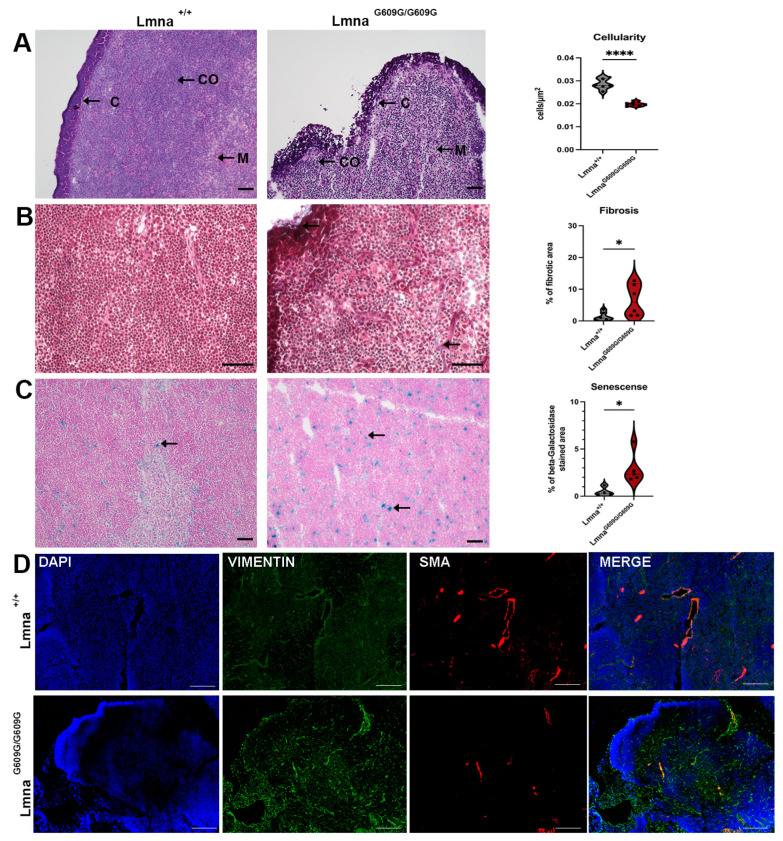
Histological and Immunofluorescent characterization of thymus pathology in Lmna^G609G/G609G^ mice compared to wildtype C57B/6 mice. Histopathological and immunofluorescent staining at 10× and 40× magnification using the Keyence BZ-X810 microscope and an Axio Imager D2 (C = capsule; CO = cortex; M = medulla): (**A**) HE staining showing significantly reduced cellularity in Lmna^G609G/G609G^ mouse thymus (n = 6; **** *p* < 0.00005; scale bar = 50 µm). (**B**) Masson’s Trichrome staining indicating increased fibrosis in Lmna^G609G/G609G^ mice, especially localized at the thymic vasculature and the thymic capsule (n = 6; * *p*< 0.05; scale bar = 50 µm; arrow = collagen deposition). (**C**) beta-Galactosidase staining of thymus indicating increased senescence in thymus (n = 3; * *p* < 0.05; scale bar = 50 µm; arrow = β-galactosidase cleaving X-Gal producing blue stain indicating senescence). (**D**) IF staining showing increased Vimentin signal in mutant mice. αSMA was reduced in Lmna^G609G/G609G^ mice (n = 3, scale bar = 100 µm).

Like the spleen, the thymus exhibited reduced size in Lmna^G609G/G609G^ mice (both organs showed approximately 40% reduction in organ weight). This size reduction was more pronounced the older the mouse was at the timepoint of death (Figure S4). Cellularity within the thymus of Lmna^G609G/G609G^ mice was significantly reduced (Figure 8A).

Masson’s Trichrome staining revealed increased collagen deposits in the interstitium and the thymus capsule (Figure 8B). Thymic retardation associated with age was suspected to be linked to increased senescence within the thymus. Indeed, we observed elevated senescence-associated beta-galactosidase positive cells in the thymic tissue of Lmna^G609G/G609G^ mice (Figure 8C). Vimentin signal was increased in Lmna^G609G/G609G^ mice, with accumulation observed in proximity to blood vessels. Furthermore, αSMA staining was reduced in the thymus of Lmna^G609G/G609G^ mice (Figure 8D), indicating a possible decreased vascularization in this primary lymphatic organ.

### 2.9. Heart

**Figure 9:**
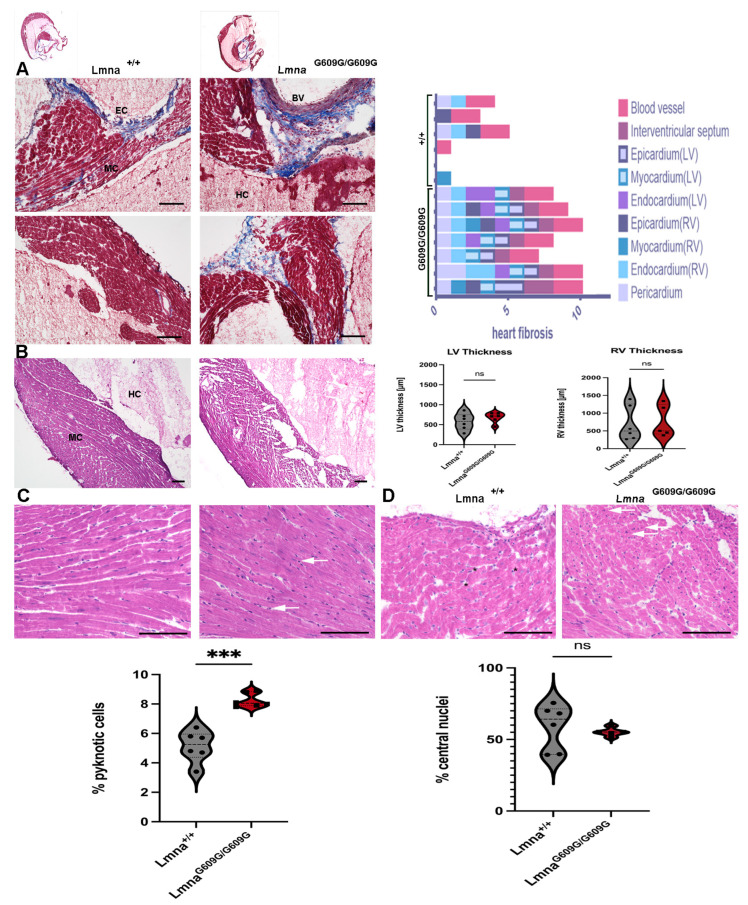
Histological and Immunofluorescent characterization of cardiac pathology in Lmna^G609G/G609G^ mice compared to wildtype C57B/6 mice (HC = heart chamber; MC = myocardium; EC = endocardium; EPC = epicardium): (**A**) Masson’s Trichrome staining indicating cardiac fibrosis. Cardiac fibrosis was observed in different cardiac regions (Blood vessel, Interventricular Septum, Epicardium, Endocardium, Myocardium, Pericardium) of Lmna^+/+^ and Lmna^G609G/G609G^ mice (n = 6 (wt)/n = 7 [25]; scale bar = 100 µm). (**B**) Left Ventricle cardiac wall thickness was measured (n = 6; *p* > 0.05 (ns), scale bar = 100 µm). (**C**) Pyknotic cells are more frequent in Lmna^G609G/G609G^ mice (n = 6; *** *p* < 0.0005, scale bar = 100 µm). (**D**) Nuclear position in cardiac myocytes was examined. Peripheral nuclei are marked by a black asterisk, central nuclei are marked by white arrows (n = 6; *p* > 0.05 (ns), scale bar = 100 µm).

To perform histopathological analysis of the heart, transverse cuts of the short axis were generated to examine both the left and right ventricles. Masson’s Trichrome staining was employed to assess fibrosis in different heart regions (Figure 9A). All Lmna^G609G/G609G^ animals exhibited increased fibrosis compared to wildtype animals (Figure 9A). Left ventricular tissues were predominantly affected in Lmna^G609G/G609G^ animals. Lmna^+/+^ mice displayed collagen deposits primarily just around blood vessels (Figure 9A). In Lmna^G609G/G609G^ mice, fibrosis was observed also in the vascular tissues, pericardium, endocardium, myocardium, epicardium, and endocardium, compartments of the heart which were less affected in Lmna^+/+^ mice (Figure 9A). However, there was no significant difference in left ventricular (LV) and right ventricular (RV) wall thickness between the two genotypes due to high variability.

A significantly increased number of pyknotic cells indicative of apoptotic cells was observed in the myocardium of Lmna^G609G/G609G^ mice (Figure 9B). Pyknotic cells are smaller and appear hyperchromatic, which is visualized by a darker hematoxylin color in those nuclei.

The number of central nuclei showed no significant difference between Lmna^G609G/G609G^ and wildtype animals (Figure 9D). Immunofluorescence of the heart revealed an increased vimentin signal in mutant animals, particularly in the epicardium and around blood vessels, while αSMA did not exhibit prominent variation between the two genotypes (Figure S10).

Collectively, the results indicate that the heart exhibits an increased level of fibrosis in the left ventricular, epicardial, and myocardial regions and shows an increased number of apoptotic cells.

## 3. Discussion

HGPS, an ultra-rare genetic pathology, serves as a remarkable model for elucidating the mechanisms behind accelerated aging processes. We offer a comprehensive overview of the pathologies observed in nine different organs of the Lmna^G609G/G609G^ mouse model of HGPS. A systematic histopathological analysis at the end of their lifespan was performed compared to wildtype littermates.

The cardiovascular system, notably the aorta, exhibited elevated collagen deposition in the media and adventitia [22]. Vascular stiffness followed by cardiovascular complications are results observed in progeria patients and the leading cause of death in this pathology.

Those aortic changes observed in this study are in accordance with previously reported observations in this HGPS mouse model [21,22]. The precise mechanism behind these pathological changes remains elusive. VSMC loss and disrupted endothelial mechanotransduction leading to the cardiovascular phenotype have been described and highlight the potential of targeting pathways involved in extracellular matrix (ECM) remodeling [28]. An increased expression of the fibrinolysis inhibitor serpine-1 (PAI-1) and Interleukin-6 (IL-6) indicated a pro-inflammatory milieu, especially in the vascular system, a phenomenon linked to aging-related cardiovascular pathologies [40]. Further, PAI-1/serpin1 has been described to induce cell-autonomous pathogenic signaling due to alterations in the nuclear lamina generated by progerin, making it a new target for therapeutics [41].

Muscular changes observed in Lmna^G609G/G609G^ mice are distinctly associated with aging-related changes in physiological aging. Notably central sarcomere nuclei, indicative of muscle damage and reduced muscular regeneration, along with a reduction in sarcomere size and increased fibrosis have been observed in this study. These findings parallel age-related muscle wasting and degeneration [42]. Increased IL-6 and Pax7 expression in Lmna^G609G/G609G^ mice indicates regenerative response and chronic inflammation in the muscle, which are two characteristics of muscle dysfunction [43,44].

In the lungs of Lmna^G609G/G609G^ mice, an increased vimentin signal was observed near the pulmonary bronchioles, associated with tissue remodeling and lung fibrosis, and indicates functional loss of the pulmonary functional unit responsible for gas exchange [35]. Senescence in the bronchiole mirrors tissue alterations known from physiological aging, leading to reduced pulmonary functionality [45].

Hepatic alterations included fibrotic changes and the reduction in hepatic fat vacuole size. Using the Ishak score [39], we characterized the level of fibrosis, observing predominantly hepato-vascular fibrosis. In the context of lipodystrophy, which is a key pathological hallmark of HGPS, we demonstrate, for the first time, a significant reduction in liver fat vacuole size in the mouse model of HGPS. The liver plays a pivotal role in lipid metabolism and hormonal processing, synthesizing various components from cholesterol and fats [46,47]. The metabolic impact of the nearly complete loss of hepatic fat warrants further investigation. Interestingly, this physiological discrepancy contrasts with physiological aging, where increased fat accumulation and the development of non-fatty liver disease (NAFLD) are observed with advanced age [48]. Another important implication of this finding is its effect on drug toxicity and retention as the FDA-approved drug for HGPS treatment, Lonafarnib, is lipophilic (Dhillon, 2021 #360) (fda.gov—Reference ID: 4705211).

Renal changes like increased vimentin signal and number of cells in glomeruli indicate an inflammatory environment. Inflammation near glomeruli and vascular fibrosis must be further investigated for their association with the renin–angiotensin pathway, as this could directly affect the cardiovascular system by increasing hypertension [49,50]. Glomerular leukocyte invasion indicates a pro-inflammatory environment in this region of the kidney, associated with aging-related nephropathic changes [51].

Splenic retardation as described previously was observed also in our Lmna^G609G/G609G^ mouse cohort [22]. Further, we were able to demonstrate collagen deposits in the spleen of vascular and interstitial localization, implicating tissue reorganization and functional changes leading to altered immune surveillance and hematopoiesis [52]. Moreover, HGPS mice show significant marginal zone (MZ) disorganization. Macrophages in the MZ are implicated in the pathogen surveillance of the blood flowing through the spleen [53]. The location of those macrophages, the MZ, generates a physical border between the white and red pulp of the spleen separating these two functionally distinct compartments of the spleen [54,55]. As we observed in the progerin mouse cohort, disorganization of the MZ suggests a reduced ability to clear blood-borne antigens and initiation of T-cell-independent immune responses [56]. In combination with the retardation, reduced cellularity, fibrosis, and senescence observed in the thymus, we provide new evidence of the potential immunological decline in this mouse model of HGPS.

Lastly, the heart displayed increased fibrosis, pyknotic cells, and elevated vimentin signal in distinct parts of the heart including the endocardium. Interestingly the regions showing the most changes in fibrosis were the vascular system and not the myocardial ones.

Our Western blot analysis showed that the Lamin A/Lamin C ratio compared to the progerin/Lamin C ratio was significantly increased in the heart and spleen. In the other organs, it was not significant but slightly decreased, except for the brain which showed a generally reduced ratio.

Collectively, we observed an increased level of fibrosis mostly in the vascular system in each organ. Interstitial fibrosis, in the heart and spleen was also increased in Lmna^G609G/G609G^ mice and was mostly prominent in the vascular system. Apart from fibrosis, we observed that every organ is affected differently by the aberrant progerin expression. Some of those changes mirror physiological aging processes such as hepatic atrophy in the liver, muscular changes in skeletal muscle, or stiffening of the aorta. While others, like the loss of hepatic lipids, are not observed during physiological aging. This comprehensive histopathology screening of Lmna^G609G/G609G^ mice at the end of their lifespan revealed widespread fibrosis and inflammation surrounding and including the vasculature system of most organs. This finding suggests that therapeutic avenues for preventing or delaying fibrosis and inflammation might provide new potential remedies targeting HGPS pathologies. Moreover, we discovered new potential targets for a possible ameliorative therapy targeting the hepatic lipid metabolism and splenic MZ organizational meshwork. A collective summary of all results for each organ is listed in Table S1.

Our study’s limitations worth noting are for one the small sample size, which might result in inadequate power potential for type I or type II error, making it possibly not applicable to larger populations. Also, just one type of mouse model might not reflect the complexity of HGPS in humans. Taking the different organ responses into a full-body context could also help to understand the pathology’s outcome. Lastly, the focus of this study is on end-stage disease which does not show inflammation or fibrosis over time as the disease progresses.

## 4. Materials and Methods

### 4.1. Mouse Model and Breeding

Transgenic Lmna^G609G^ mice were kindly provided by Carlos-Lopes Otin, Departamento de Bioquimica y Biologia Molecular, University of Oviedo (Spain). The mice carrying the HGPS phenocopy mutation have been generated and described earlier [22]. Mouse breeding and housing were conducted with the permission of the Bavarian state government according to the Animal Welfare Act. The colony was initiated via embryo transfer to secure SPF grade pathogen-free animal husbandry according to the Federation of Laboratory Animal Science Association (FELASA) following the recommendations for health monitoring of rodent and rabbit colonies in the Breeding and Experimental Units Act from 2002. To minimize inbreeding, a minimum of five distinct mouse families were maintained at any given time. After three mating generations, Lmna^G609G/+^ males were mated with 8-week-old C57BL/6 female mice (027C57BL/6; Charles River, Sulzfeld, Germany). The mice were fed standard chow (PS RM-H, V1534; ssniff Spezialdiäten GmbH, Soest, Germany) and housed under a 12 h light/dark cycle at controlled temperatures (21–22 °C) and humidity (50%). Environmental enrichment such as red a plexiglass tunnel, red house, cotton balls, and wood chippings provided a species-appropriate environment. Mice were housed separated by sex, with a maximum of 5 mice in one cage. Maintenance breedings were conducted by co-housing one Lmna^+/+^ female and one Lmna^G609G/+^ male from 8 weeks until 20 weeks of age (or until 24 weeks in cases without litter and subsequent change of heterozygous males). Lmna^G609G/+^ male and female offspring from maintenance breedings were used to generate Lmna^G609G/G609G^ mice. Homozygous animals were exported to scientists for the conduction of experiments and monitoring in an SPF-grade experimental animal husbandry facility with similar light, temperature, and humidity parameters as in the maintenance area. Lmna^G609G/G609G^ animals were provided with extra litter and cotton for nest-building and were co-housed with at least one Lmna^+/+^ or Lmna^G609G/+^ littermate to prevent hypothermia. From the age of 8 weeks, these animals were given water-soaked chow.

### 4.2. Genotyping

Genetic material was extracted from earmark punches obtained at weaning. The extraction was performed using a homemade DNA extraction kit with the Mixing Block MB-102 (BIOER, Hangzhou, China) at 95 °C. PCR was conducted using published primers [22] and amplification as described previously [21] using the Bio-RAD iCycler thermocycler.

### 4.3. Mouse Organ Harvest and Sample Preparation

Experimental animals were euthanized by cervical dislocation under 5% isofuran anesthesia. The mice were shaved and perfused with 20 mL PBS (Sigma Aldrich, St. Louis, MO, USA). Organs for Western blotting were collected and immediately snap-frozen. Organs used for histopathological analysis were placed in cryo-molds (Sakura Tissue-Tek Cryomold #4565, Torrance, CA, USA) according to organ size and mounted in OCT (Sakura Tissue-Tek O.C.T Compound #4583). After OCT was applied, organs were frozen using liquid nitrogen. Tissue orientation in OCT was standardized (heart apex down, skin standing vertically in OCT, and organ harvest side were always from the same location (e.g., for muscle, only gastrocnemius/quadriceps was collected). Samples were stored at −80 °C until further processing.

### 4.4. Histological Staining

Histopathology was conducted on 6 µm thick tissue sections prepared using a Leica cryotome (LEICA CM3050S, Wetzlar, Germany). Histopathology was evaluated using alizarin red, Oil Red O (Sigma Aldrich, #O0625-25G), H&E (abcam, ab245880, Cambridge, UK), beta-gal, and Masson’s Trichrome (abcam, AB150686) stainings. Staining protocols were performed according to manufactures’ protocols. Tissue-specific adjustments and detailed staining protocol have been further described (Supplementary Methods S1). We performed the histological analysis on samples from 12 different animals (6× Lmna^+/+^ and 6× Lmna^G609G/G609G)^.

### 4.5. Immunofluorescent Staining

Immunocytochemistry was performed on 6 µm thick cryotome cuts (see histology staining). Tissue sections were fixed for 10 min at −20 °C in methanol. After a wash with PBS, sections were permeabilized with 0.2% triton X-100 in PBS for 30 min and washed with PBS. Slides were then blocked for 1 h in PBS supplemented with 10% FBS. Incubation with the primary antibodies diluted in 10% FBS was performed at room temperature for a specified period of time (Table 1).

The secondary antibodies used were affinity purified Alexa Fluor^®^ 555 or 488 conjugated anti-rabbit/mouse/goat antibodies (Life Technologies, Carlsbad, CA, USA, A21206 anti-rabbit-488, A21202 anti-mouse-488, A31572 anti-rabbit-555, and A31570 anti-mouse-555, 1:600) (Table 2).

After primary antibody incubation, slides were rinsed 4 times with 20% FBS in PBS for 30 s each. The second antibody was then applied for 1 h at room temperature. Following antibody incubation, slides were washed twice in 20% FBS for 5 min and twice in PBS for 5 min. Next, DAPI Vectashield mounting medium was applied (Vector Laboratories, VEC-H-1200, Newark, CA, USA). Images were acquired using the Keyence BZ-X810 system.

### 4.6. Western Blot Analysis

Mouse tissues were homogenized using stainless steel beads and a Dounce homogenizator (Carlroth CXE1.1, Karlsruhe, Germany). Protein concentration was estimated using a Bradford assay. BSA was used as a standard (BioRad Laboratories, Hercules, CA, USA, 5000206). Gel electrophoresis was performed followed by protein transfer on a nitrocellulose membrane. The membrane was blocked for 1 h with 5% non-fat milk and incubated overnight at 4 °C with antibodies depicted in Table 3 (Table 3).

After primary antibody incubation, the membrane was washed three times with TBS-T for 5 min. After washing, the corresponding secondary antibody was applied for 1 h at RT. Visualization of the immunoblot was performed with a ChemiDoc^TM^ MP. Quantification of Western blot data was performed with Biorad ImageLab 4.1 software. Normalization was performed on total protein and GAPDH.

### 4.7. Statistical Analysis

All experiments have been conducted in at least three biological replicates. Statistical analysis was performed using R studio or Graphpad Prism 7. Significance between different groups was tested using the Student *t*-test and ordinary regression analysis. Significance was displayed as * *p* < 0.05, ** *p* < 0.005, and *** *p* < 0.0005.

## Figures and Tables

**Table 1:** Overview table primary antibodies.

Antibody ID	Company	Ref #	Incubation Time	Dilution
Lamin A/C	Proteintech (Martinsried, Germany)	81042-1-RR	ON	1:250
αSMA	MERCK (Saint Louis, MO, USA)	C6198-100UL	ON	1:1000
IL-8	Antibodies.com (Stockholm, Sweden)	A26910	ON	1:500
Il-6	Invitrogen (Carlsbad, CA, USA)	P620	ON	1:1000
P16/NK4a	Invitrogen (Carlsbad, CA, USA)	MA5-17142	ON	1:500
PAI-1/serpine-1	Invitrogen (Carlsbad, CA, USA)	MA5-17171	ON	1:500
CD68	Abcam (Boston, MA, USA)	Ab283667	ON	1:500
Vimentin	Cell Signalling (Danvers, MA, USA)	D21H3	ON	1:500

**Table 2:** Overview table secondary antibodies.

Antibody ID	Company	Ref #	Incubation Time	Dilution
Alexa Fluor 555(mouse)	Invitrogen (Carlsbad, CA, USA)	A31570	1 h	1:1000
Alexa Fluor 488(mouse)	Invitrogen (Carlsbad, CA, USA)	A21202	1 h	1:1000
Alexa Fluor 555(rabbit)	Invitrogen (Carlsbad, CA, USA)	A31572	1 h	1:1000
Alexa Fluor 488(rabbit)	Invitrogen (Carlsbad, CA, USA)	A21206	1 h	1:1000

**Table 3:** Overview table primary and secondary antibodies for Western Blot Analysis.

Antibody ID	Company	Ref #	Incubation Time	Dilution
αSMA	MERCK (Saint Louis, MO, USA)	C6198-100UL	ON	1:2000
Lamin A/C	Santa Cruz (Santa Cruz, CA, USA)	Sc-20681	ON	1:5000
Peroxidase AffiniPure Goat Anti-Rabbit IgG (H + L)	Jackson Immuno Research (West Grove, PA, USA)	111035003	1 h	1:5000
Peroxidase AffiniPure Goat Anti-Mouse IgG (H + L)	Jackson Immuno Research (West Grove, PA, USA)	115035003	1 h	1:5000

## Data Availability

Data are contained within the article and Supplementary Materials.

## Supplementary Materials

The following supporting information can be downloaded at: https://www.mdpi.com/article/10.3390/ijms25179323/s1.
