# Supplementary material for: Inflammation and Fibrosis in Progeria: Organ-Specific Responses in an HGPS Mouse Model
Source: Int J Mol Sci. 2024 Aug 28;25(17):9323. doi: 10.3390/ijms25179323 (PMC11395088; doi:10.3390/ijms25179323)
Supplement: Supplementary file 1 [file ijms-25-09323-s001.zip › ijms-3176975-supplementary.pdf]

International Journal of
Molecular Sciences

MDPI

Supplementary Materials:

Supplementary Methods – Histology Staining Protocols

S1: Histology Staining Protocols + Tissue-specific modifications

Alizarin Red:

Tissue sections were fixed in 4% paraformaldehyde (PFA) 10 minutes, and stained with Alizarin Red solution for 30 seconds to highlight calcium deposits. After staining, excess dye was removed by gently shaking off the slides, and the sections were blotted dry. The samples were then dehydrated by immersion in acetone for 2 minutes, followed by a clearing step using a 1:1 mixture of acetone and xylene for another 2 minutes. Subsequently, the slides were cleared in 100% acetone for 1 minute and mounted with a synthetic resin.

Oil Red O:

Tissue sections were fixed in 4% paraformaledehyde (PFA), in the meantime 150mg of ORO (Oil Red O) powder was solved in 50ml 99% Isopropanol. The solution was filtered using a filter paper. The filtrate functions as the stock solution. We continued to make a new solution with 60% stock solution and 40% MilliQ water. This new solution was filtered again and we received the working solution. After tissue sections were fixed in PFA we rinsed them with MilliQ water and put them inside 60% isopropanol for 3 minutes. Tissues samples were transferred to ORO working solution and incubated for 10 minutes. After the staining slides were put in 60% isopropanol and the vessel was inverted 5 times. Sections were taken out and blotted dry for 2 minutes. Hematoxylin counterstaining was applied for 2 minutes (always prepare freshly). Slides were rinsed shortly in MilliQ. After this we rinsed slides under tap water or used bluing reagent for 1 min. Slides were blotted dry and mounted with 9:1 gycerol:PBS.

Hematoxylin & Eosin Staining:

Tissue sections were fixed in 4% paraformaledehyde (PFA) for 10 minutes and stained with hematoxylin for 2 minutes. Slides were rinsed shortly with MIlliQ water and put inside blueing reagent for 20 seconds. Slides were rinsed again with MIlliQ water and incubated in two changes of 100% ethanol for 1 minute. Slides were dipped 2x in Eosin Y solution for 5 seconds each time. Slides were then rinsed with MilliQ water and dehydrated in four changes of ethanol (50% - 70%-95%-100%) each for 1 minute. After dehydration sections were transferred to Xylene for 8 seconds. Slides were left to dry and then mounted by synthetic resin.

Note:

4x dip in Eosin for Aorta

Trichome Staining:

Bouin's Fluid was preheated to 54-64°C. Tissue sections were fixed in 2% paraformaledehyde (PFA) for 10 minutes and incubated in preheated Bouin's Fluid for 30 minutes. Slides were taken out of Bouin's Fluid and left at room temperature to cool down for 10 minutes. Sections where then rinsed under running tap water until yellow color was completely gone. Slides were put into MilliQ water shortly and immediately transferred to Hematoxylin for incubation for 5 minutes.

Slides were put under running tap water for 2 minutes. After blueing slides were transferred to Bieberich Scarlet/ Acid Fuchsin for 5 minutes. Slides were rinsed after this with MilliQ water and differentiated in 5% phosphomolybdic- / phosphothungistic acid for 10 minutes. Without washing slides were placed in Anilin Blue staining solution for 3 minutes. We rinsed the slides again in MIlliQ water and applied 0.5% acetic acid to the sections for 3 minutes. Slides were dehydrated in three changes of ethanol (70%-90%-100%) for 1 minute each and transferred to xylene for 1 minute. Slides were dried and mounted with synthetic resin.

Note:

- **for muscle and heart the slides should be incubated overnight in Bouins fluid at room temperature**
- **scarlet fuchsin should be applied for 10 min in muscle and heart**

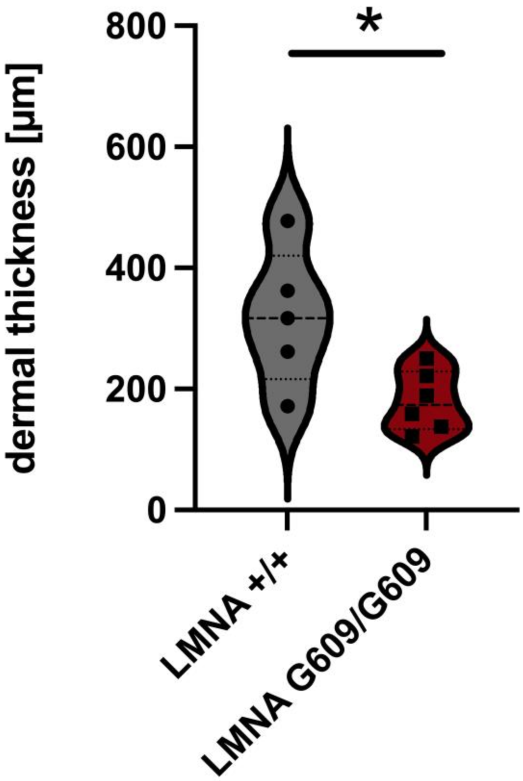

Figure S1. Dermal thickness comparing $Lmna^{+/+}$ and $Lmna^{G609G/G609G}$ histological cuts(+/+; n = 5; G609G/G609G; n = 6).

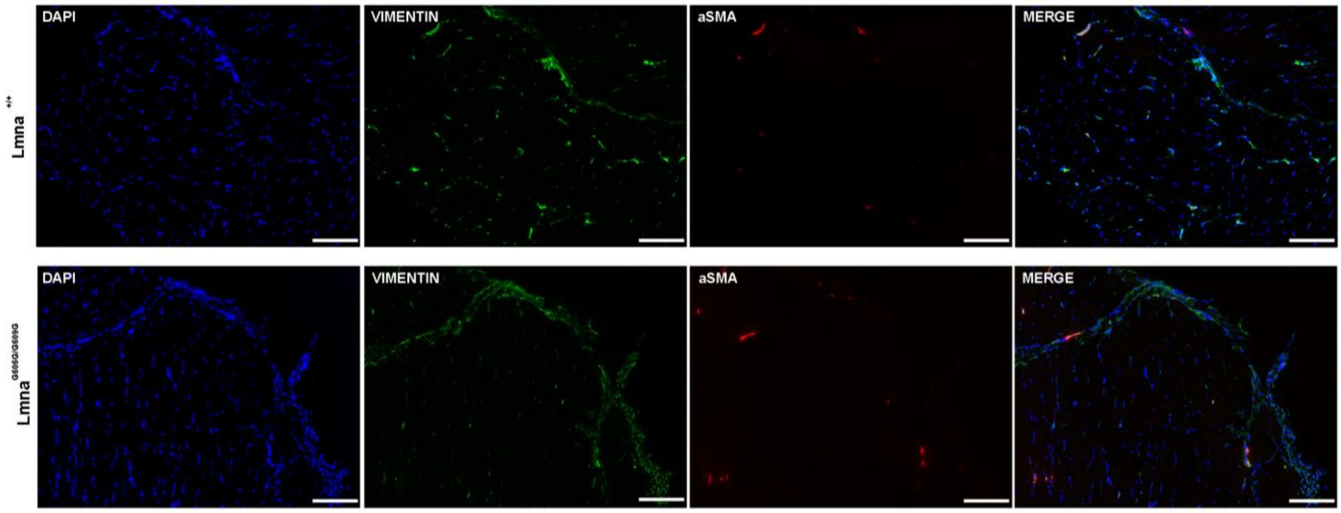

Figure S2. Immunofluorescent staining of Lmna$^{+/+}$ and Lmna$^{G609G/G609G}$ muscle tissue using vimentin(green) and αSMA [34] antibodies (scale bar = 200μm).

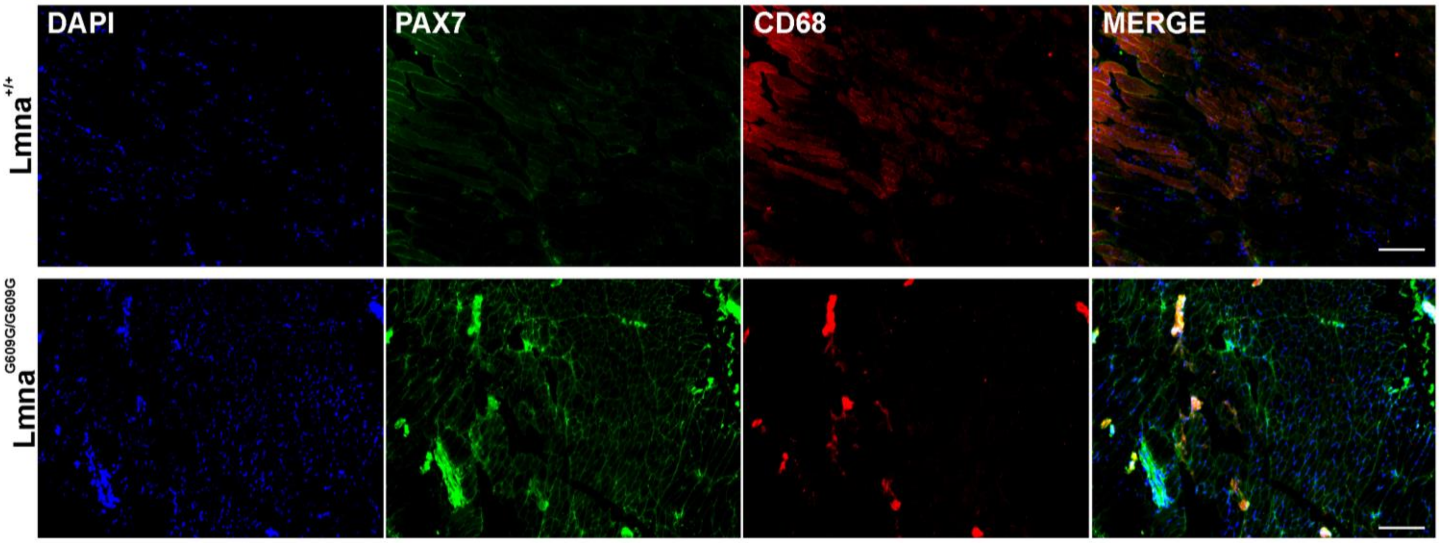

Figure S3. Immunofluorescent staining of Lmna$^{+/+}$ and Lmna$^{G609G/G609G}$ muscle tissue using Pax7(green) and CD68 [34] antibodies (scale bar = 200μm).

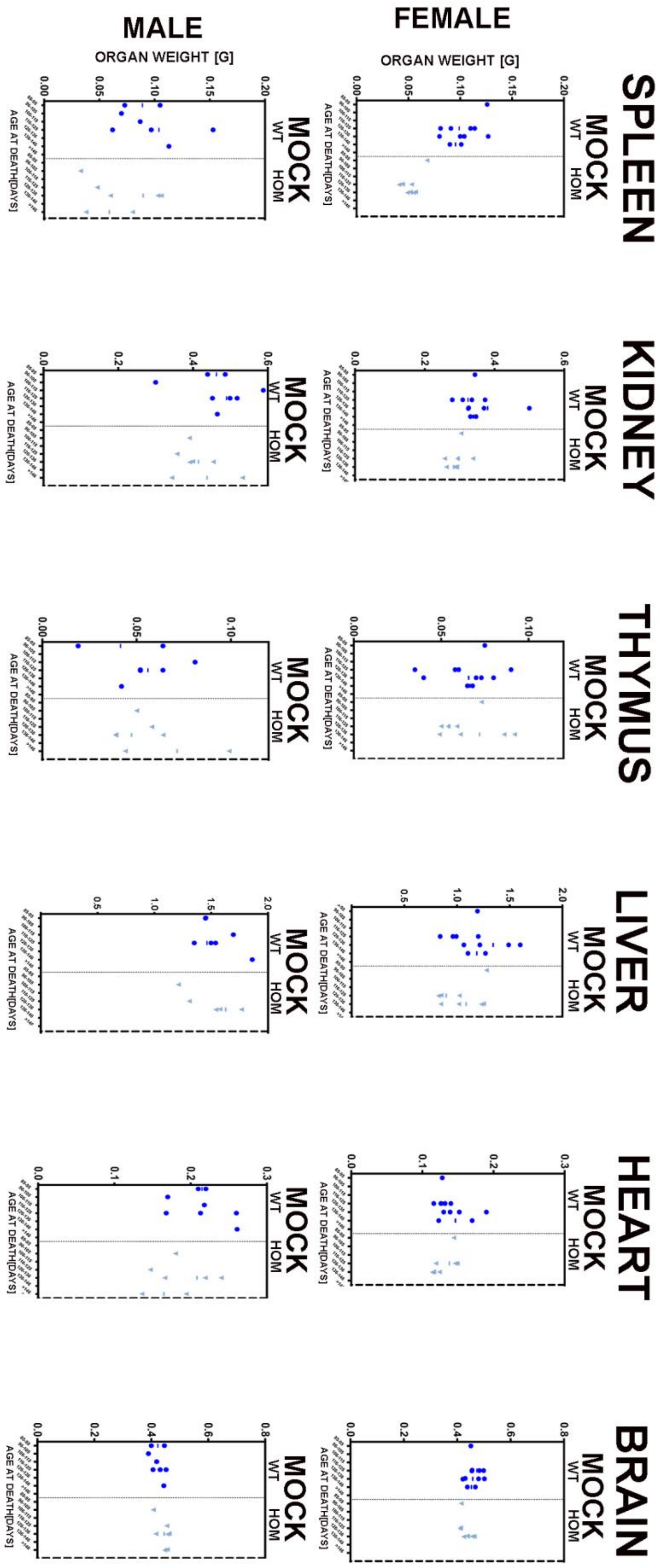

Figure S4. Mouse organ weight plottet against the age of death(days). We compare +/+ and G609G/G609G mice.

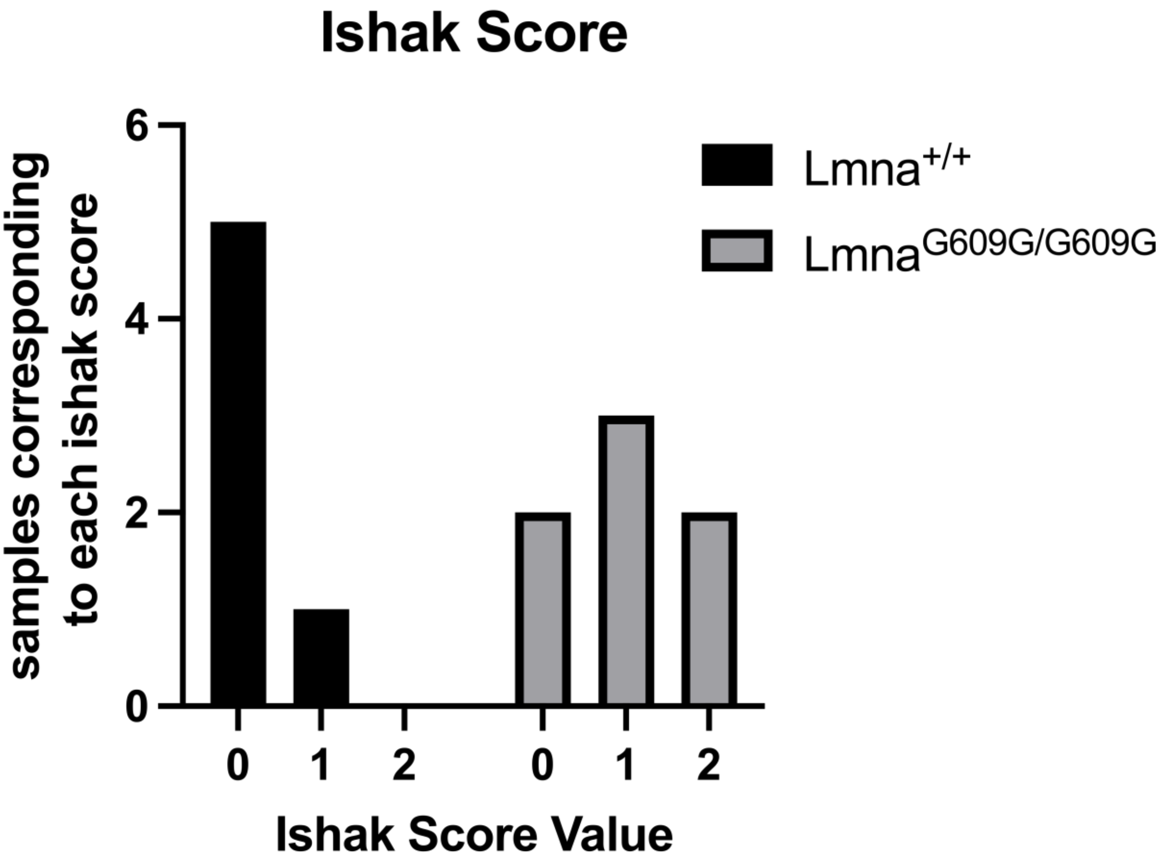

Figure S5. Ishak score accounted for each sample. In +/+ mice the score was mostly 0 indicating no signs of fibrosis. In the G609G/G609G most of the samples scored 1 or 2 indicating slight to mediocre fibrosis levels mostly around vasculature and minimal in the hepatic interstitium.

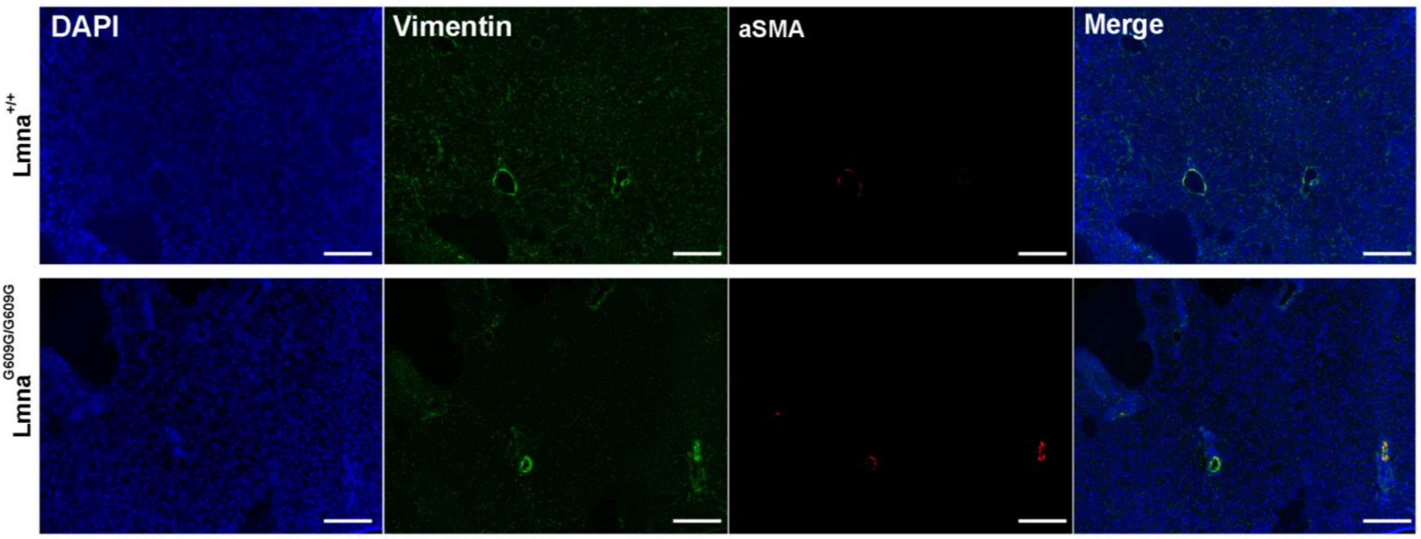

Figure S6. Immunofluorescent staining of Lmna$^{+/+}$ and Lmna$^{G609G/G609G}$ liver using anti-Vimentin (green) and anti-aSMA antibodies (scale bar = 200μm).

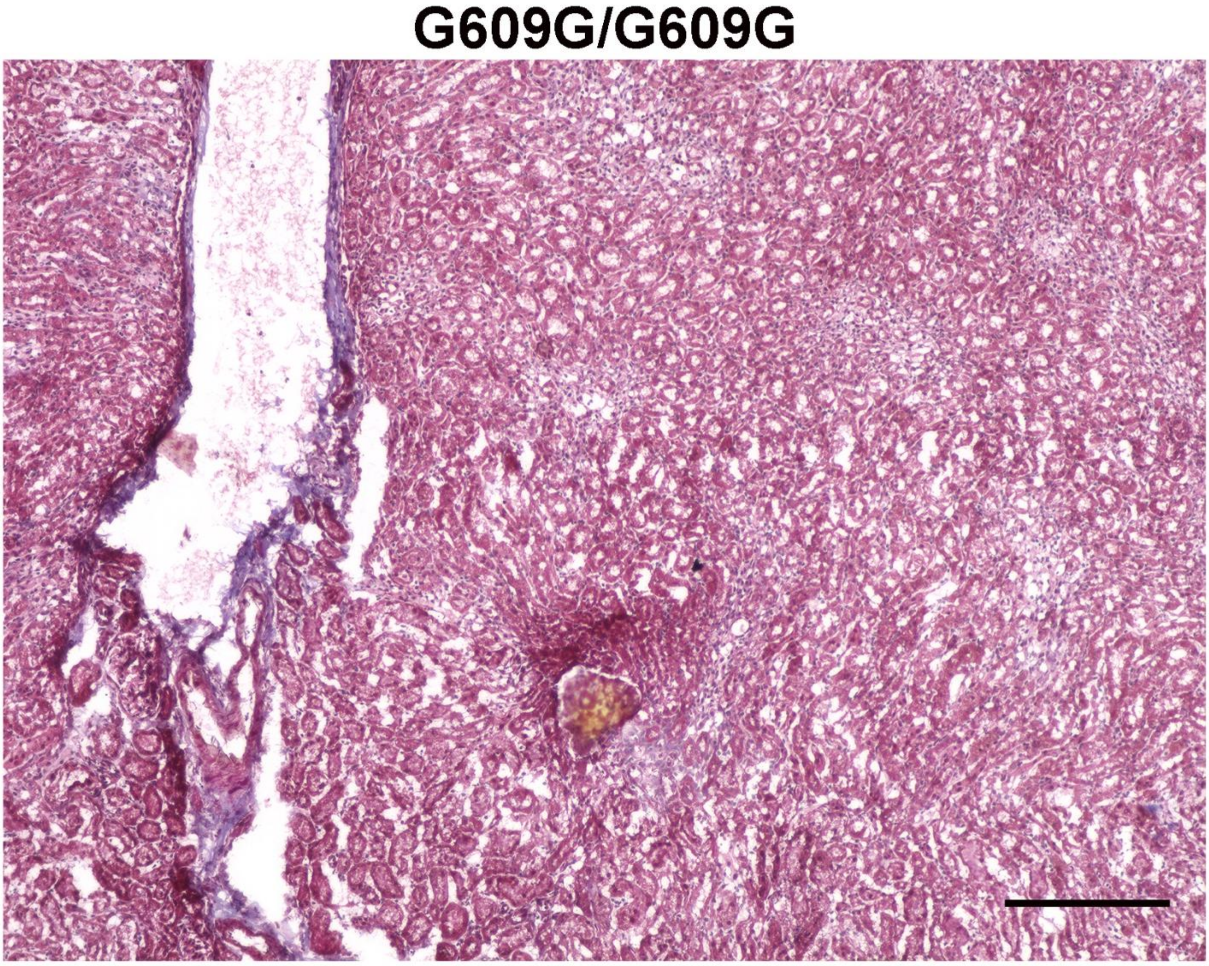

Figure S7. Trichome staining of G609G/G609G kidney depicting crystalline debris. Only once observed in mutant mice (scale bar = 200μm)

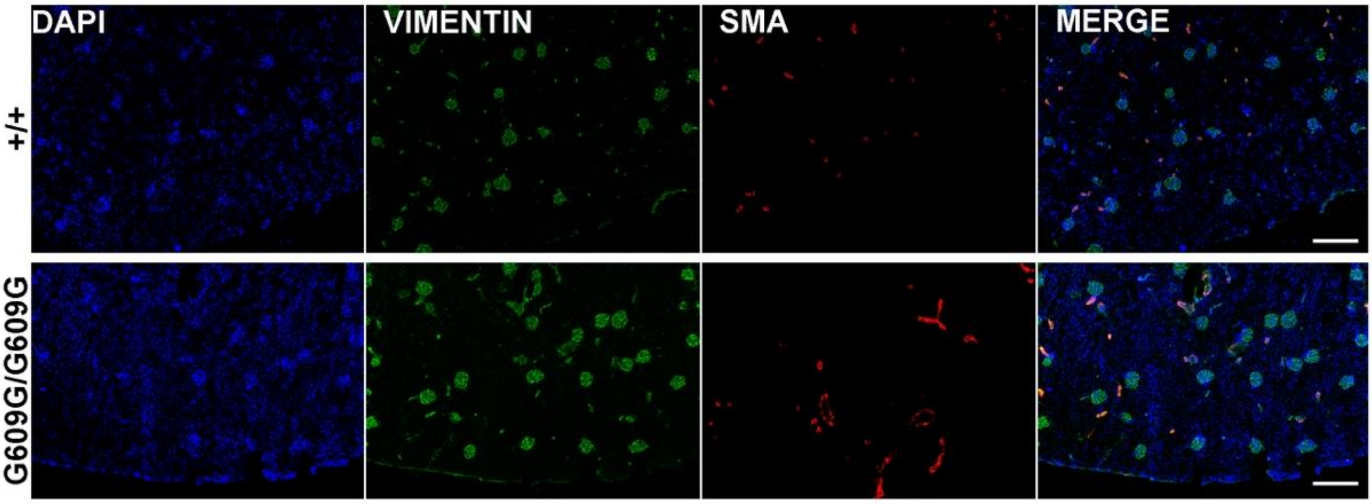

Figure S8. Immunofluorescent staining of Lmna$^{+/+}$ and Lmna$^{G609G/G609G}$ kidney using anti-Vimentin (green) and anti-aSMA antibodies (scale bar = 100μm).

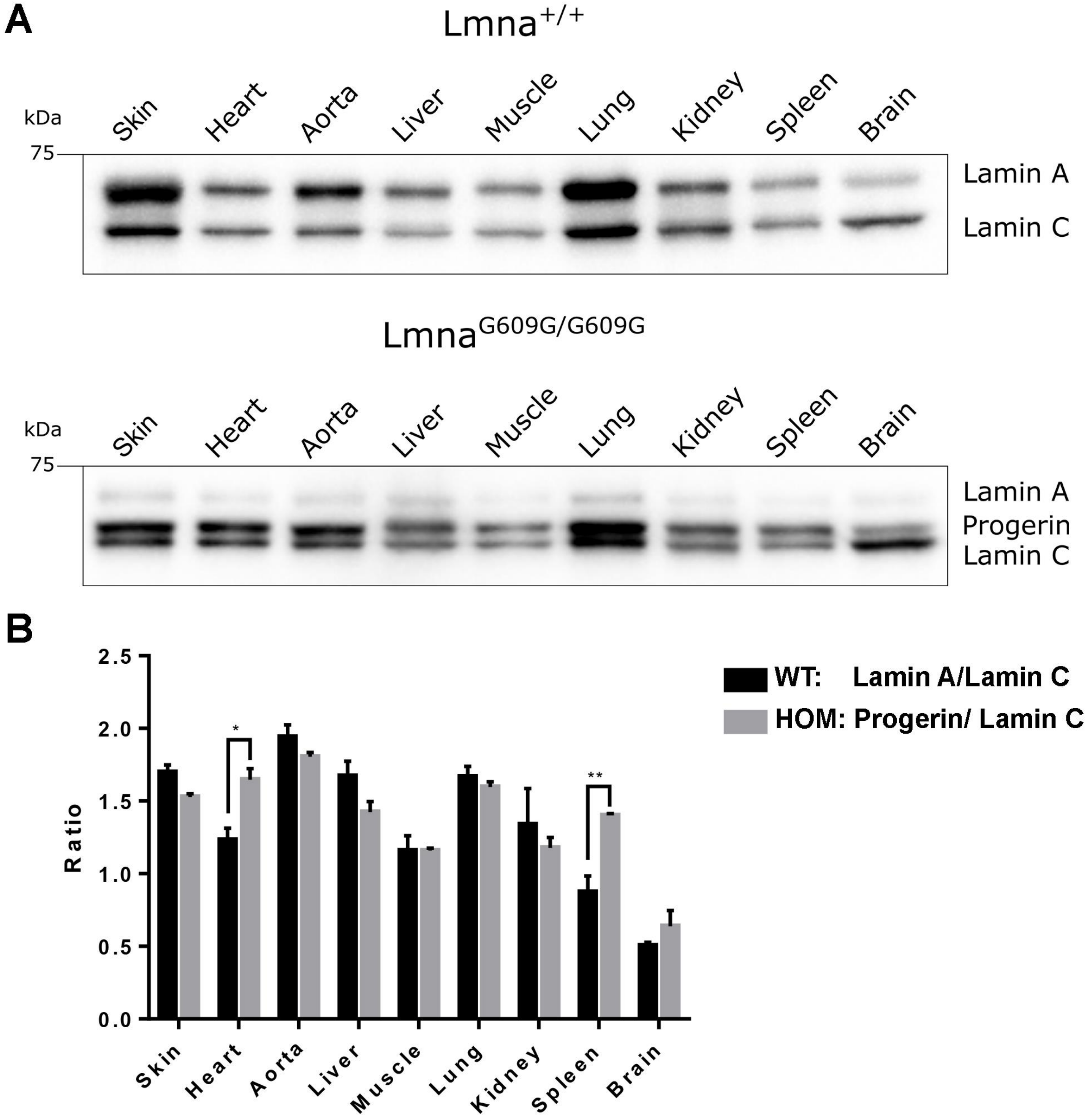

Figure S9. Western blot analysis comparing Lamin A/Lamin C in $Lmna^{+/+}$ and progerin/ Lamin C in $Lmna^{G609G/G609G}$ expression in all organs (n = 3). B: Calculation of Lamin A/ Lamin C ratio in $Lmna^{+/+}$ mice comparing with progerin/ Lamnin C ratio in $Lmna^{G609G/G609G}$ mice in all organs (n = 3; p<0.05 = *, p<0.01 = **). The ratios remained mostly similar across the genotypes for all organs except for heart and spleen in which we observed an increase in the Progerin levels indicating progerin accumulation.

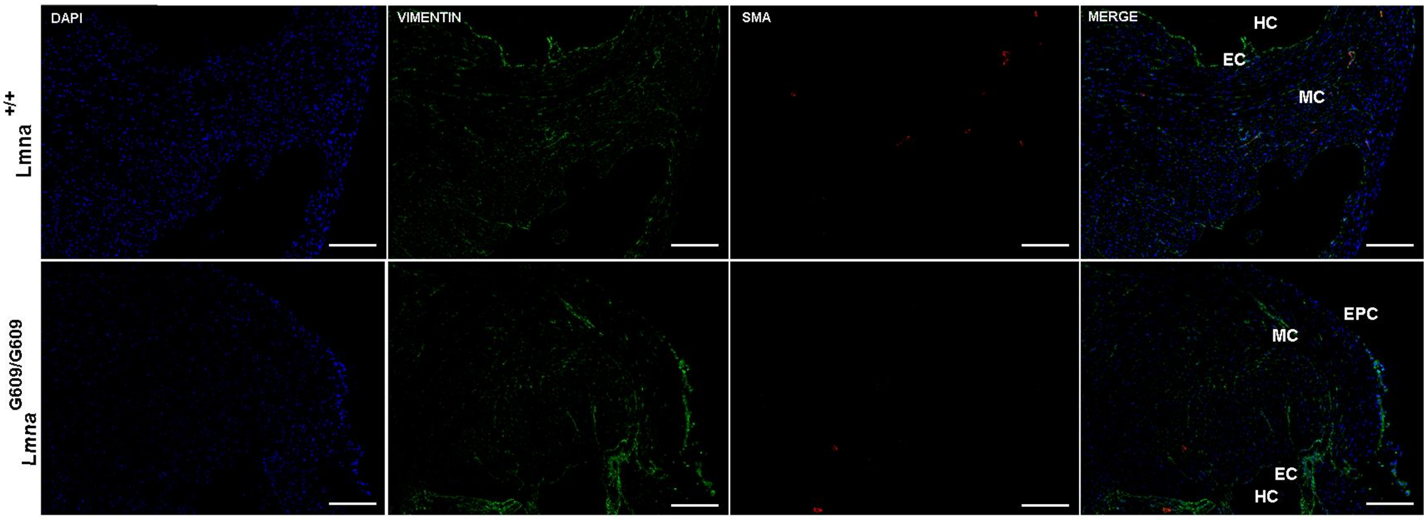

Figure S10. Immunofluorescent staining of Lmna$^{+/+}$ and Lmna$^{G609G/G609G}$ heart tissue using Vimentin(green) and aSMA [34] antibodies (scale bar = 200μm).

organ	histopathological evaluation	biomarker	conclusion for Lmna$^{G609G/G609G}$ mice
**skin**	- reduced dermal cellularity<br>- increased dermal fibrosis<br>- reduced skin thickness (males)	p16	increased levels in dermis and hypodermis
		Vimentin	decreased vimentin expression in areas of dermal layer<br>increased vimentin signal in hypodermal layer
		PAI-1/serpine-1	increased levels in dermis accumulating in focused regions (beacons)
		IL-6	(no changes observed)
**aorta**	- increased media and adventitia fibrosis<br>- significant VSMC loss in media<br>- elastin fiber dysruption<br>- reduced media thickness	Lamin A/C	showing nuclear lamina composition
		αSMA	Strongly reduced indicating VSMC loss
		p16	elevated signal in the endothelial layer of the aortic intima
		Vimentin	Depletion of vimentin in media
		PAI-1	Increased levels in the aortic intima and adventitia
		IL-6	marked increase in the aortic media indicating strong inflammation
**muscle**	- reduced sarcomere cross-section size	Pax7	increased levels in sarcomeric periphery
		CD68	(no changes observed)

	- increased number of central nuclei<br>- elevated levels of muscle tissue fibrosis	αSMA	(no changes observed)
**lung**	- increased beta-galactosidase staining around club cells of the bronchiole suggesting elevated levels of senescence in this tissue area<br>- bronchiolar and vascular fibrosis increased	Vimentin	elevated levels of vimentin found in lung tissue indicating hyperactivation and proliferation of fibroblasts and enhanced collagen synthesis
		PAI-1	increased levels at bronchioles
		IL-6	(no changes observed)
**liver**	- reduced fat vesicle size and therefore depletion of hepatic fat stores<br>- increased cellularity – reduced overall cell size in disease model<br>- increased levels of fibrosis with prominent portal fibrosis but no observed fibrosis of the hepatic sptum	Bodipy	liver fat vesicles smaller indicating reduced fat deposits in liver
**kidney**	- reduction in kidney cortex diameter and overall kidney size<br>- increased number of cells in glomeruli<br>- no rim enlargement around glomeruli<br>- significant increase in collagen deposition around vasculature of the renal system<br>- no observation of renal duct or interstitial fibrosis of the kidney<br>- indication of calcifications	P16	strongly increased levels of expression in glomeruli
		Vimentin	increased signal at the rim of the glomerolus
		PAI-1	glomeruli exhibit increased signal of PAIL-1
		IL-6	(no changes observed)
	- observable reduction in marginal zone organization – WP rim disorganized	Vimentin	increased signal in red pulp aligning with the histopathological findings of interstitial fibrosis

**spleen**	- elevated levels of interstitial fibrosis<br>- increased levels of vascular fibrosis<br>- unchanged total ratio of WP and RP<br>- generally decreased size of spleen	αSMA	loss of VSMC in splenic vasculature
		P16	(no changes observed)
**thymus**	- pronounced size reduction<br>- reduced thymic cellularity<br>- increased beta-galactosidase signal detected indicating increased levels of senescence	Vimentin	increased signal with elevated amounts in blood vessel proximity
		αSMA	reduced amounts detected indicating reduced vascularization
**heart**	- increased fibrosis detected predominantly affecting left ventricle, vasculature, pericardium, epicardium, myocardium and endocardium<br>- increased amount of pyknotic cells indicating cell apoptosis<br>- no difference in central nuclei	Vimentin	increased signal found in epicardium and around blood vessels
		αSMA	(no changes observed)

Table S1: Summary result table listing each organ with the corresponding histopathological analysis and biomarker findings.
